# Neural-behavioral dissociation under acute high-altitude stress: an exploratory ERP study of non-specific neural recruitment and rTMS effects

**DOI:** 10.3389/fnbeh.2026.1844235

**Published:** 2026-06-19

**Authors:** Peng Wu, Lizhi Liu, Huanhuan Li, Jiangheng Guan, Yang Lu, Xianglin Kong, Jian Song, Guozheng Xu

**Affiliations:** 1 Department of Neurosurgery, Central Theater General Hospital of Chinese PLA, Wuhan, China; 2 Hubei Provincial Clinical Medical Research Center for Minimally Invasive Treatment of Cerebrovascular Diseases, Wuhan, China; 3 Department of Neurosurgery, Changjiang River Shipping General Hospital, Wuhan, China; 4 School of Medicine, Wuhan University of Science and Technology, Wuhan, China; 5 School of Medicine, Hubei University of Medicine, Shiyan, China; 6 School of Medicine, Southern Medical University, Guangzhou, China

**Keywords:** cognitive function, conflict processing, event-related potentials (ERPs), flanker task, hypobaric hypoxia, neural compensation, repetitive transcranial magnetic stimulation (rTMS)

## Abstract

**Objective:**

Acute high-altitude hypobaric hypoxia impairs executive functions, yet the dynamic relationship between behavioral performance and neuroelectric activity remains unclear. This study aimed to explore the neural compensatory mechanisms underlying cognitive dysfunction induced by high-altitude stress and to conduct an exploratory investigation of the potential modulatory effects of repetitive transcranial magnetic stimulation (rTMS).

**Methods:**

Thirty-one healthy adults completed a Flanker task while undergoing EEG recording at three time points in a repeated-measures design: at baseline (G1), immediately after 24 h of simulated high-altitude hypobaric hypoxia (3,600 m; G2), and after three consecutive days of active 1 Hz repetitive transcranial magnetic stimulation (rTMS) applied to the right dorsolateral prefrontal cortex (DLPFC) (G3). We analyzed behavioral (reaction time, accuracy) and event-related potential (N2, P3 amplitude) outcomes. Importantly, we calculated conflict effect difference scores (ΔN2, ΔP3) to isolate neural activity specific to conflict processing.

**Results:**

Reaction times were significantly faster at G2 and G3 than at G1 (*p* < 0.001), with accuracy remaining stable. N2 and P3 amplitudes were significantly enhanced at G2 and G3 relative to G1 (all *p* < 0.05). ΔN2 did not differ across time points, whereas ΔP3 showed a transient increase from G1 to G2 (*p* < 0.01) and returned to baseline at G3. As an exploratory study without a sham control, causal interpretations are limited, but the findings reveal clear dissociation between overall neural activity and conflict-specific processing.

**Conclusion:**

Acute high-altitude stress increases neural activity related to conflict monitoring and attentional allocation. This enhancement reflects non-specific neural recruitment rather than improved specificity in conflict processing. Exploratory observations indicate that rTMS further elevates P3 amplitude without selectively modulating conflict-specific responses. Conflict effect scores (ΔN2, ΔP3) show promise as sensitive markers for evaluating neurocognitive states under hypoxic stress.

## 1 Introduction

Acute high-altitude hypobaric hypoxia significantly impairs higher-order cognitive functions, such as conflict monitoring and executive control (Chen et al., 2017; Falla et al., 2021; Su et al., 2024). The Flanker paradigm has been shown to reliably detect hypoxia-related changes in cognitive processing, including alterations in reaction times and response consistency (Bliemsrieder et al., 2022). These behavioral changes are accompanied by disruptions in neural energy metabolism and neurotransmitter system function (Wang et al., 2022; Coronel-Oliveros et al., 2024).

However, the dynamic relationship between behavioral impairments and cognitive-related neuroelectrical activity remains to be fully understood. Event-related potentials (ERPs) offer a distinctive insight into these mechanisms (Duncan et al., 2009; Walla, 2025; Yüce et al., 2026). Specifically, the N2 component, which originates from the anterior cingulate cortex (ACC), is indicative of conflict monitoring (Chen et al., 2025), while the P3 component is associated with the allocation of attentional resources (Forster et al., 1975). An increase in N2/P3 amplitudes following a challenge may indicate compensatory neural recruitment (Ortiz-Prado et al., 2025; Taylor et al., 2025). Nonetheless, it remains unclear whether this enhanced neural response constitutes an adaptive functional adjustment or an inefficient mobilization of cognitive resources, a phenomenon known as “inefficient compensation.”

Traditional ERP studies have primarily focused on changes in absolute amplitude, interpreting these changes as direct indicators of functional status. However, recent advancements in cognitive neuroscience suggest that the intensity and efficiency of neural activity are distinct dimensions. Neural efficiency is defined as the brain’s capacity to achieve comparable behavioral performance using fewer neural resources.

The CRUNCH model, proposed by Reuter-Lorenz and Cappell, suggests that the brain engages additional resources to sustain functionality when task demands surpass neural capacity, but this recruitment is subject to physiological limitations, and excessive recruitment may paradoxically impair processing efficiency (Reuter-Lorenz and Cappell, 2008). Agbangla et al. (2019) have further explored the phenomenon of “inefficient processing,” where certain individuals can successfully activate task-relevant brain regions, yet this activation does not translate into behavioral benefits, resulting in enhanced neural activity without corresponding improvements in behavior. In the context of conflict monitoring research, neural efficiency can be quantified by examining the differences in neural activity between incongruent and congruent conditions (Wang et al., 2014).

Accordingly, the current study introduces conflict effect sizes (ΔN2, ΔP3) as indices of neural efficiency to isolate the additional neural resources specifically engaged in conflict processing (see Methods for detailed definitions). This approach offers a more sensitive measure of resource utilization efficiency under stress (Clayson and Larson, 2011; Jiang et al., 2013).

Transcranial magnetic stimulation (TMS) has the potential to modulate the function of specific brain networks (Liu et al., 2025). While repetitive TMS (rTMS) has been promising in cognitive rehabilitation, its effectiveness in ameliorating acute hypoxia-induced cognitive impairments has not yet been conclusively demonstrated. Importantly, empirical evidence is still required to determine whether TMS can optimize the potentially aberrant relationship between neural activity and behavioral performance under conditions of stress.

Grounded in the theoretical framework of neural efficiency, this study utilized 1 Hz rTMS targeting the right dorsolateral prefrontal cortex (DLPFC) to investigate its potential to improve the neuro-behavioral relationship under conditions of high-altitude stress. The rationale for selecting 1 Hz rTMS targeting the right DLPFC is supported by several lines of evidence: First, clinical efficacy: Randomized controlled trials and meta-analyses have consistently shown that 1 Hz rTMS applied to the right DLPFC effectively reduces anxiety symptoms (Mantovani et al., 2013; Sasaki et al., 2013; Lefaucheur et al., 2020; Liu et al., 2023), which is pertinent since exposure to high altitudes frequently triggers anxiety, potentially disrupting cognitive control networks. Second, mechanistic alignment: The right DLPFC, a central component of the executive control network, plays a crucial role in conflict monitoring and attention allocation (Duan et al., 2023). Its classical inhibitory effect on cortical excitability (Rossi et al., 2009) has the potential to modulate abnormal activation patterns induced by hypoxia (Gong et al., 2025). Third, evidence of neuroplasticity: Five consecutive days of 1 Hz rTMS have been observed to enhance dendritic complexity and foster neuronal development (Cambiaghi et al., 2020). Although longer protocols (e.g., 5 days) are commonly used in clinical studies targeting chronic conditions, the acute nature of high-altitude stress (24-h exposure) and the need to capture immediate post-intervention effects within a constrained experimental window favored a shorter regimen. Notably, our pilot data confirmed that three consecutive sessions at 40% RMT were sufficient to elicit reliable changes in the targeted ERP measures, thereby minimizing participant burden while preserving sensitivity to detect the modulatory effects on neural efficiency.

Therefore, this study aimed to determine whether 1 Hz rTMS targeting the right DLPFC could optimize the neuro-behavioral relationship under acute high-altitude stress by suppressing aberrant neural activity and fostering neuroplastic changes.

The research employed a repeated-measures design, integrating 24-h simulated high-altitude (3,600 m) hypobaric hypoxia exposure with task-state EEG and rTMS interventions. Behavioral performance and neuroelectrophysiological activity were assessed at three time points: baseline (G1), immediately post-exposure (G2), and post-rTMS (G3). Neural efficiency was quantified using conflict effect sizes (ΔN2/ΔP3) to isolate the neural resources engaged in conflict processing. Changes in the N2 and P3 components were analyzed to determine whether the rTMS intervention could mitigate the inefficient neural compensation state induced by high-altitude stress.

## 2 Materials and methods

### 2.1 Participants

The G*Power 3.1 software (Faul et al., 2007) was utilized to calculate the required sample size. For a repeated-measures analysis of variance (ANOVA) with a medium effect size (Cohen’s *f* = 0.25), α = 0.05, and power (1-β) = 0.80, specified for the within-subject interaction effect (time × condition), it was estimated that a minimum of 28 participants would be required. To account for an expected dropout rate of approximately 30% (e.g., due to excessive EEG artifacts or failure to complete all sessions), 40 healthy right-handed volunteers were recruited.

Between March 2024 and January 2025, 40 university students were recruited through public advertisements. All 40 recruited participants met the inclusion criteria; none were excluded for failing to meet these criteria. Following a data quality screening, 9 participants were excluded due to poor EEG signal quality, defined as: (1) excessive artifacts (e.g., muscle tension, eye blinks, or movement) remaining after independent component analysis (ICA) cleaning, (2) fewer than 80% of valid trials retained per condition after artifact rejection, or (3) incomplete recording sessions. The remaining 31 participants (24 males, 7 females; mean age 24.00 ± 2.45 years) successfully completed the full protocol and were included in the final analysis. All participants possessed normal or corrected-to-normal vision, had no history of neurological or psychiatric disorders, no cardiovascular or respiratory diseases, and no history of residing or traveling to high-altitude regions (above 2,500 m).

The inclusion criteria were as follows: (1) aged between 18 and 35 years; (2) having a bachelor’s degree or higher; (3) right-handedness; (4) absence of smoking or alcohol abuse; (5) a Mini-Mental State Examination (MMSE) score of 27 or higher prior to the experiment; (6) a Montreal Cognitive Assessment (MoCA) score of 26 or higher prior to the experiment; and (7) willingness to comply with all experimental procedures. The exclusion criteria included: (1) a history of craniotomy or severe head trauma; (2) a history of psychiatric or neurological disorders; (3) contraindications to rTMS (e.g., metal implants, epilepsy history); (4) use of medications affecting central nervous system function within the previous 3 months; and (5) inability to cooperate with electrophysiological evaluation.

### 2.2 Ethics statement

This study was approved by the Ethics Committee of the General Hospital of the Central Theater Command of the Chinese People’s Liberation Army (Approval No. [2024] 008-01). All participants provided written informed consent prior to participation, and all procedures were conducted in accordance with the Declaration of Helsinki.

### 2.3 Experimental design

**FIGURE 1:**
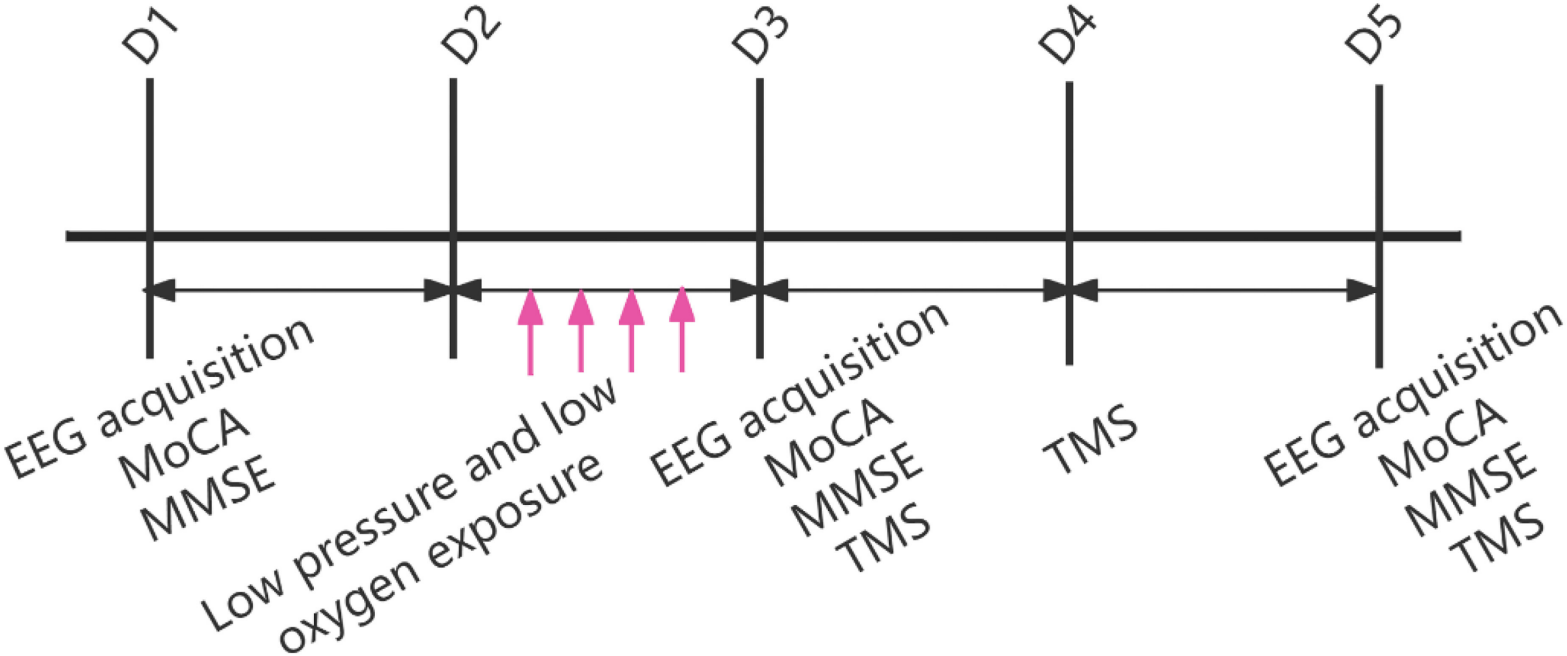
Schematic illustration of the experimental procedure and assessment time points. Pink arrow: onset of hypobaric hypoxia exposure. D1: baseline (G1). D2–D3: 24-h exposure. D3 also: post-exposure assessment (G2) and first rTMS. D4: second rTMS. D5: third rTMS and final assessment (G3).

The present study utilized a prospective, single-group, repeated-measures design. Participants sequentially underwent five distinct phases: baseline assessment (G1), 24-h hypobaric hypoxia exposure, immediate post-exposure assessment (G2), three consecutive days of repetitive transcranial magnetic stimulation (rTMS) intervention, and final post-intervention assessment (G3). At three critical time points, G1, G2, and G3, participants completed the MoCA and the MMSE, followed by EEG recordings during the Flanker task. We analyzed behavioral indicators such as RT and accuracy, along with ERP components (N2 and P3 amplitudes), to elucidate the dynamic changes in cognitive function throughout the experimental phases (refer to Figure 1). EEG recordings were performed at standardized time points: G2 was acquired within 3 h after participants exited the hypobaric chamber, and G3 was acquired within 30 min after the third and final rTMS session. All participants exited the hypobaric chamber simultaneously. Immediately afterward, a trained team of experimenters assisted participants in EEG cap placement and preparation, utilizing two synchronized EEG acquisition systems running in parallel.

### 2.4 Hypoxia exposure protocol

**FIGURE 2:**
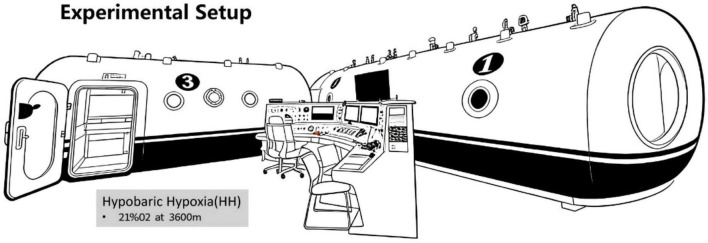
Schematic diagram of the experimental setup for simulated high-altitude hypobaric hypoxia exposure.

A hypobaric chamber (Model YC-3600J) simulated a high-altitude hypoxic environment at an elevation of 3,600 m (see Figure 2). Chamber conditions were meticulously regulated: barometric pressure was maintained at 483 ± 5 mmHg (mirroring the atmospheric pressure at 3,600 m), and oxygen concentration was sustained at 20.34 ± 0.5%, resulting in an inspired oxygen partial pressure of approximately 96 mmHg. The chamber’s temperature was consistently held between 22 and 24°C, with relative humidity controlled between 50 and 60% to ensure participant comfort and to eliminate potential confounding effects from variations in temperature and humidity. Although continuous physiological monitoring (SpO2, heart rate) was not performed in this study, the hypobaric hypoxia environment was strictly controlled and maintained at a stable altitude of 3,600 meters throughout the 24-h exposure, in accordance with standardized protocols for simulated high-altitude studies.

Participants remained in the chamber for a continuous 24-h period. During this time, to mitigate the additional effects of physical activity on physiological and cognitive measures, participants were restricted to sedentary activities such as reading, watching videos, or using electronic devices in a non-task-related manner. Regular meal and sleep schedules were maintained. The entire exposure process was continuously supervised by trained research personnel, who ensured the stability of environmental parameters and the safety of the experimental conditions. The 24-h duration was selected because previous studies indicate that acute hypobaric hypoxic exposure induces significant neurophysiological alterations (e.g., a rise in white matter cerebral blood flow) at this time point (McGuire et al., 2019).

### 2.5 TMS intervention protocol

Immediately following their exit from the hypobaric chamber, participants received three consecutive days of rTMS treatment. Resting Motor Threshold (RMT) was determined following international guidelines before the first rTMS session. With participants awake and fully relaxed, RMT was defined as the minimum intensity producing a detectable cortical motor response in at least 5 out of 10 consecutive stimuli. Stimulation intensity was set to 40% of individual RMT (Rossi et al., 2009). The stimulation was administered using a YRD CCY-I magnetic stimulator equipped with a standard 70 mm figure-of-eight coil, targeting the right DLPFC. Localization was based on the international 10–20 EEG system, specifically at the F4 electrode position.

The stimulation parameters were set as follows: low-frequency stimulation at 1 Hz, with an intensity of 40% of the resting motor threshold (RMT). Each stimulation train comprised 30 pulses, with an inter-train interval of 3 s. A total of 34 trains were delivered daily, totaling 18 min and 42 s per session and 1,020 pulses per day. The coil was positioned tangentially to the scalp surface, angled backward at approximately 45° to the sagittal plane to optimize the direction of the induced current. All sessions were conducted at consistent times each day by trained personnel, who also closely monitored the participants’ subjective sensations and immediate responses throughout the intervention.

Notably, the present study employed a relatively low rTMS intensity of 40% RMT, which was selected based on the following rationales with strong literature support. First, low-intensity rTMS (30–50% RMT) is sufficient to elicit reliable neurophysiological changes without inducing excessive cortical excitability or discomfort (Fuggetta et al., 2008; Lefaucheur et al., 2020), as subthreshold stimulation can modulate synaptic efficacy, neural circuits, and functional connectivity through calcium signaling and metabolic pathways rather than relying on suprathreshold motor activation (Tang et al., 2021; King and Tang, 2024). Second, acute hypobaric hypoxia is known to increase cortical excitability; conventional high-intensity rTMS (80–120% RMT) may carry a substantial risk of overactivation and network disruption in this vulnerable state, whereas low-intensity rTMS offers a safer and more physiologically appropriate strategy (Moretti and Rodger, 2022). Third, in clinical and cognitive studies involving sensitized brain states, low-intensity rTMS has been repeatedly verified to induce measurable and robust changes in ERP components (including N2 and P3) that reflect cognitive control processes (Moretti et al., 2022; Wang et al., 2024); our pilot data further confirmed that 40% RMT was adequate to elicit stable ERP changes without adverse events. Fourth, mounting evidence demonstrates that stimulation intensity does not need to exceed motor threshold to achieve meaningful neuromodulatory effects, especially for non-motor targets such as the DLPFC involved in cognitive control (Lefaucheur et al., 2020; Tang et al., 2021). Therefore, 40% RMT was adopted as a safe, rigorously justified, and physiologically suitable intensity for exploratory neuromodulation under acute high-altitude stress. Our rTMS protocol (1 Hz, 40% RMT, right DLPFC, 3 days) was chosen based on recent evidence regarding low-intensity neuromodulation for cognitive control. This protocol aligns with safety and efficacy guidelines for non-invasive brain stimulation in healthy individuals under stress challenge (Ebrahimzadeh et al., 2025b; Ebrahimzadeh and Soltanian-Zadeh, 2026).

### 2.6 Flanker task paradigm

**FIGURE 3:**
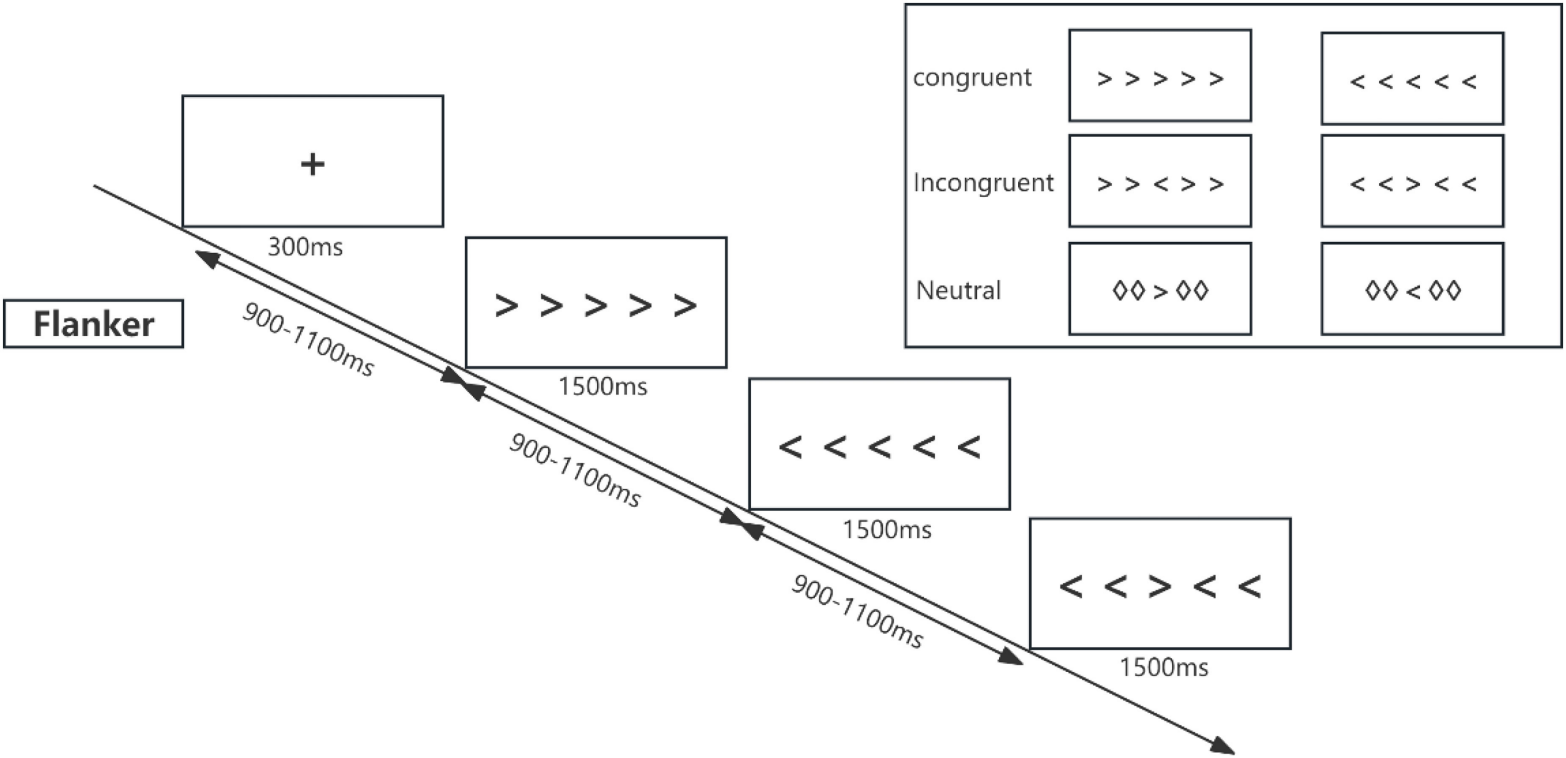
Schematic illustration of the Flanker task paradigm. Each trial began with a fixation cross “ + ” for 300 ms (as shown), followed by a 1,500 ms display of five arrows. Three conditions: neutral (diamond flankers), congruent (same direction), incongruent (central arrow opposite). Participants pressed left/right mouse button to indicate central arrow direction. ISI: 900–1,100 ms. Total of 180 trials in three blocks.

All participants performed a modified version of the Eriksen flanker task. This task was conducted using E-Prime 2.0 software (Psychology Software Tools, Inc., Sharpsburg, PA, United States) on a computer screen. As depicted in Figure 3, the flanker paradigm comprised five horizontally aligned arrows forming three stimulus types: (1) neutral—where the central arrow was flanked by a diamond; (2) congruent—with all arrows pointing in the same direction (e.g., “→→→→→” or “←←←←←”); and (3) incongruent—where the central arrow pointed opposite to the flanking arrows (e.g., “→→←→→” or “←←→←←”).

Participants undertook the task in a semi-dark, quiet room, positioned approximately 80 cm from the monitor, which displayed a white background and black stimuli. They were instructed to disregard the flanking stimuli and to respond as quickly and accurately as possible to the direction of the central arrow by pressing the left or right mouse button (with hand-response mapping counterbalanced among participants). Each trial proceeded as follows: a central fixation cross “ + ” appeared for 300 ms to ensure a stable baseline; the target stimulus was then presented for 1,500 ms; the response window was defined as 1,500 ms from stimulus onset, during which participants were required to respond. After stimulus offset, a blank screen appeared with a random inter-stimulus interval ranging from 900 to 1,100 ms to prevent temporal expectancy (Duncan et al., 2009; Kanske and Kotz, 2010).

The formal experiment consisted of three blocks, each comprising 60 trials, resulting in a total of 180 trials, with an equal distribution of the three stimulus types (neutral, congruent, incongruent) within each block. These were presented in a pseudo-random order within each block. A practice block of 20 trials preceded the formal experiment to ensure participants fully understood the task requirements. EEG signals were recorded simultaneously during the task, which lasted approximately 10–12 min.

### 2.7 EEG data acquisition

Continuous EEG signals were captured using a 64-channel Ag/AgCl electrode cap (ANTNeuro, Enschede, Netherlands) connected to an eego mylab amplifier. The placement of electrodes followed the international 10–20 system. The CPz electrode functioned as the online reference, and the AFz electrode served as the ground. CPz was chosen because it is located at the midline centro-parietal region, which is relatively stable and less susceptible to ocular and muscle artifacts compared to mastoid electrodes. AFz was selected as the ground to minimize frontal polar artifacts. The sampling rate was 1,000 Hz, and a hardware bandpass filter of 0.5–100 Hz was applied during acquisition to avoid aliasing and to preserve high-frequency information for subsequent offline filtering. Impedances for all electrodes were maintained below 10 kΩ before initiating data collection.

During the recording sessions, experimenters vigilantly monitored the ongoing EEG traces to identify and exclude segments contaminated by ocular, muscular, or movement artifacts. All recordings were conducted inside a shielded room to minimize external electromagnetic interference. The selected acquisition parameters conformed to the prevailing standards in task-related ERP research.

### 2.8 EEG data preprocessing and time-domain analysis

Offline EEG data processing was conducted using the EEGLAB toolbox (version 2019.0) on MATLAB R2022b (MathWorks, Natick, MA, United States). The analysis pipeline consisted of several consecutive stages.

Initially, raw continuous data were loaded, and electrode coordinates were established in accordance with the international 10–20 system. Channels that did not record scalp activity, including electrooculography (HEOG, VEOG) and mastoid electrodes (M1, M2), were excluded, resulting in 60 channels available for further analysis. To reduce low-frequency drifts and remove high-frequency noise, a bandpass filter of 0.5–30 Hz was applied, followed by a 48–52 Hz notch filter to further eliminate residual power-line interference. Although the 30 Hz low-pass filter provides partial attenuation of 50 Hz noise, the notch filter was additionally applied to ensure complete removal, given the high sensitivity of ERP analyses to line-frequency artifacts.

Subsequently, the continuous signal was segmented into epochs that were time-locked to the delivery of stimuli, extending from 200 ms prior to 800 ms following stimulus onset. Channels with poor signal quality were identified through visual inspection combined with statistical criteria. Specifically, a channel was flagged as defective if it met any of the following conditions: (1) its standard deviation exceeded 3 times the median standard deviation of all channels; (2) its correlation coefficient with the average of its neighboring channels fell below 0.4; or (3) its amplitude range exceeded ± 200 μV. Such channels were reconstructed using spherical spline interpolation. The number of interpolated channels per participant did not exceed six, representing fewer than 10% of the total channels. Epochs containing significant artifacts, such as those induced by head movements or muscle contractions, were manually excluded, ensuring that at least 80% of trials per condition were preserved for each participant.

Following this, independent component analysis (ICA) was applied using the Infomax algorithm as implemented in EEGLAB (Bell and Sejnowski, 1995). Artifactual components (e.g., blinks, saccades, muscle tension, cardiac artifacts) were identified based on four predefined criteria: (1) typical frontal or lateral-frontal scalp topography; (2) transient high-amplitude deflections in the time course; (3) dominant power below 5 Hz (ocular artifacts) or above 20 Hz (muscle artifacts); and (4) blind visual inspection and consensus by two experienced researchers. All identified artifactual components were removed before further preprocessing. After ICA cleaning, the data were re-referenced to the average of all channels.

**FIGURE 4:**
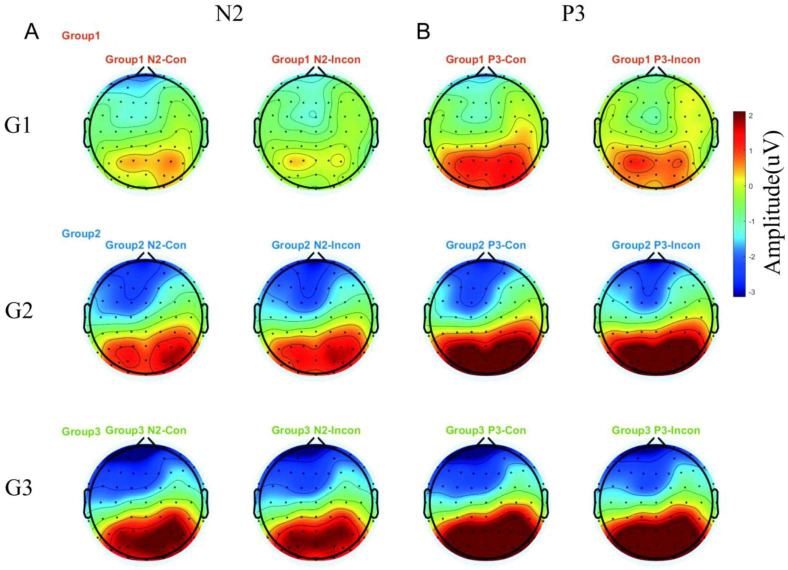
Scalp topographies of N2 and P3 components across time points and task conditions. **(A)** N2 topographies at three time points (G1, G2, G3) under congruent (Con) and incongruent (Incon) conditions. **(B)** P3 topographies at the same time points (G1, G2, G3) under congruent (Con) and incongruent (Incon) conditions.

**FIGURE 5:**
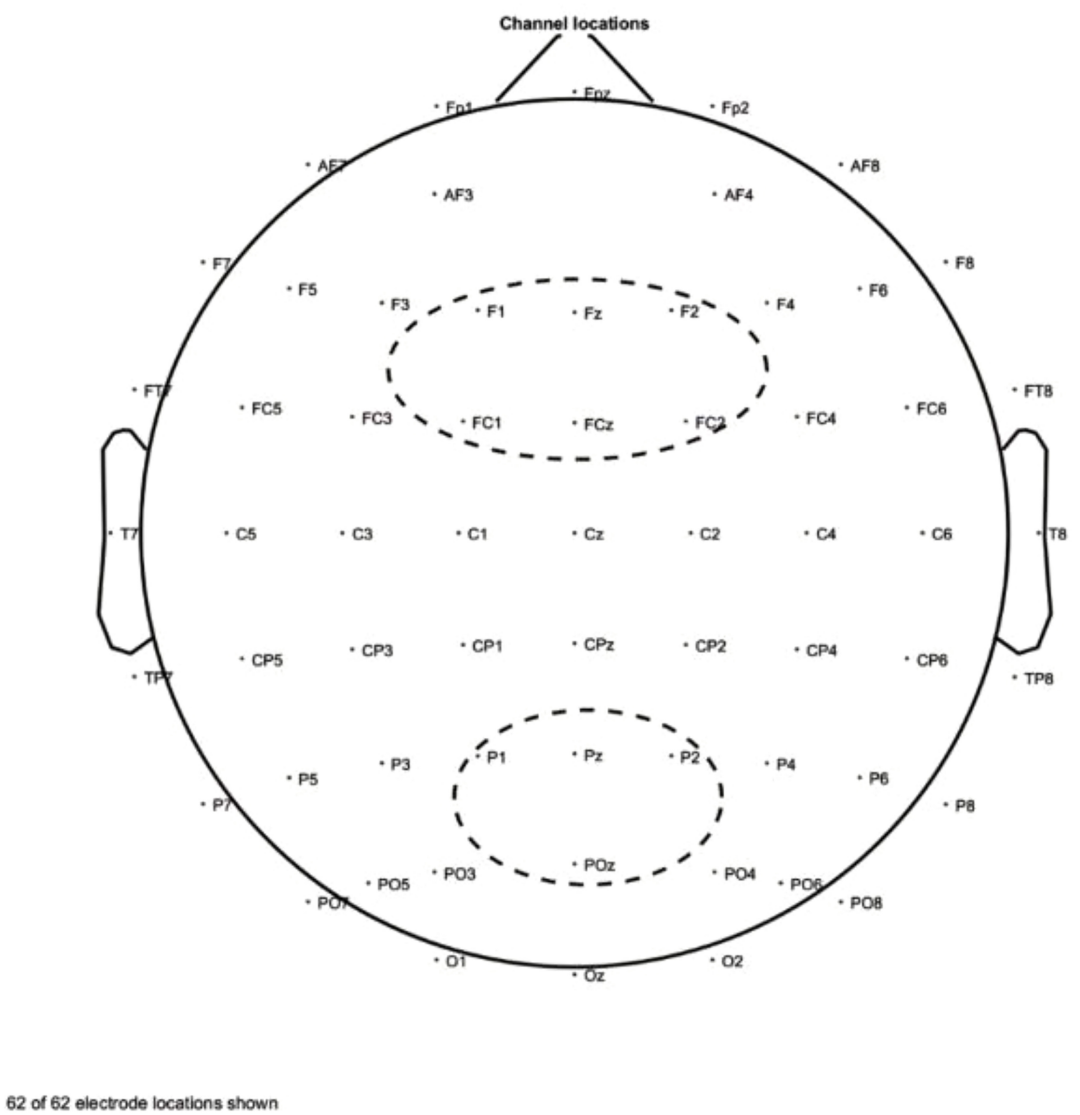
Regions of interest for N2 and P3 components on scalp topography. Dashed circles indicate the electrode clusters selected for each component. The N2 component was analyzed at six frontocentral electrodes (F1, Fz, F2, FC1, FCz, FC2). The P3 component was analyzed at four parietal electrodes (P1, Pz, P2, POz).

For each participant, artifact-free epochs were averaged separately across time points (G1, G2, G3) and experimental conditions (congruent, incongruent). Baseline correction was applied using the pre-stimulus interval from −200 to 0 ms. After artifact rejection, all included participants retained > 80% of trials per condition. Detailed trial retention statistics are provided in Supplementary Table S3. The mean trial count was not calculated, as retention below this threshold led to exclusion. Informed by the grand-averaged waveforms and existing literature, the N2 component was quantified as the mean amplitude within the 250–320 ms window at each of six frontocentral electrodes (F1, Fz, F2, FC1, FCz, FC2). Similarly, the P3 component was quantified as the mean amplitude within the 250–300 ms window at each of four parietal electrodes (P1, Pz, P2, POz). Separate repeated-measures ANOVAs were then conducted for each electrode to preserve topographical specificity. This early window, selected based on the grand-averaged waveforms, precedes the classic P3b (300–600 ms) and likely reflects an early attentional subcomponent (e.g., P3a). Under the average reference used, this parietal positivity cannot be a mathematical sign-flip of the frontal N2: the two components show distinct scalp topographies (frontocentral vs. parietal; see Figure 4) and opposite temporal dynamics (negative-going N2 vs. positive-going P3) within the overlapping time window, ruling out a dipolar sign-flip artifact. The early time window of 250–300 ms was chosen based on the prominent peak observed in our grand-averaged waveforms. This window captures the early P3a subcomponent related to initial attentional allocation and conflict evaluation, rather than the classic later P3b component. This justification has been clarified to enhance methodological rigor. The chosen electrode groupings align with the typical scalp distributions of each component, as illustrated in Figure 5. Our ERP extraction and difference wave analysis followed validated pipelines as recommended in previous studies. The use of ΔN2 and ΔP3 to isolate conflict-specific neural activity is consistent with established approaches for conflict processing assessment (Ebrahimzadeh et al., 2013).

### 2.9 Statistical analysis

For the behavioral data, trials with incorrect responses or RTs that exceeded ± 3 standard deviations from the mean for each individual were excluded from the analysis. Subsequently, the mean RT and accuracy (ACC) were calculated for each participant across three time points (G1, G2, G3) and under two task conditions (congruent, incongruent).

The Shapiro–Wilk test confirmed that all dependent variables (reaction time, accuracy, N2 amplitude, P3 amplitude) were normally distributed across conditions and time points (all *p* > 0.05). Separate repeated-measures analyses of variance (ANOVAs) were conducted for the behavioral measures (RT, ACC) and ERP measures (N2 amplitude, P3 amplitude), using time point (G1, G2, G3) and condition (congruent, incongruent) as within-subject factors. This method facilitated the examination of the main effects of time and condition, as well as their interaction.

To further quantify conflict-specific neural activity during conflict processing, conflict effect sizes were calculated as follows:

ΔRT = RTincongruent–RTcongruent

ΔACC = ACCincongruent–ACCcongruent

ΔN2 = N2 amplitude (incongruent) –N2 amplitude (congruent)

ΔP3 = P3 amplitude (incongruent) –P3 amplitude (congruent)

These difference scores isolate the neural activity specifically engaged in conflict processing by subtracting the baseline activity of congruent trials from that of incongruent trials. A smaller difference score may indicate relatively reduced recruitment of additional neural resources for conflict resolution, whereas an unchanged or larger difference score—despite stable or improved behavioral performance—may suggest relatively increased or non-specific neural recruitment (Reuter-Lorenz and Cappell, 2008; Clawson et al., 2017; Agbangla et al., 2019).

Difference wave analysis (incongruent minus congruent) is a classic approach in ERP research for quantifying conflict-related neural activity. By subtracting the neural activity of congruent trials from that of incongruent trials, these difference scores isolate the additional neural resources engaged specifically in conflict processing, serving as markers of conflict-specific neural activity.

Additionally, separate one-way repeated-measures ANOVAs with time point (G1, G2, G3) as the within-subject factor were conducted on ΔN2 and ΔP3 to directly assess changes in conflict processing efficiency over time.

For all ANOVAs, Greenhouse-Geisser corrections were applied when the assumption of sphericity was violated. Significant main effects or interactions were further explored through *post-hoc* comparisons using the Bonferroni correction. Effect sizes are reported as partial eta-squared (η^2^). To ensure transparency and readability, simplified tables are presented in the main text, while complete descriptive and statistical data are provided in Supplementary Tables S1, S2.

## 3 Results

### 3.1 General information and scale scores

The final analysis included thirty-one healthy participants (24 males, 7 females), with ages ranging from 20 to 31 years (mean 24.0 ± 2.5 years). Regarding educational attainment, 21 participants (67.7%) had obtained or were pursuing a bachelor’s degree, while the remaining 10 participants (32.3%) had obtained or were pursuing a master’s degree or higher.

A repeated-measures ANOVA revealed a significant main effect of time on MoCA scores [*F*(2, 60) = 3.1, *p* = 0.05, η^2^ = 0.09]. *Post-hoc* comparisons indicated that MoCA scores at G2 (29.4 ± 1.1) were significantly lower than those at G1 (29.7 ± 0.6, *p* = 0.04). Scores at G3 (29.8 ± 0.4) returned to baseline levels and did not significantly differ from G1 (*p* = 0.21). For MMSE scores, no significant effect of time was observed [*F*(2, 60) = 1.7, *p* = 0.19, η^2^ = 0.05], indicating that global cognitive function remained stable across all experimental phases (Table 1).

**TABLE 1:** General information and scale scores.

	Value/frequency	Proportion (%)	Statistic (*F*/χ^2^)	*P*-value
Age (years)				
Range	20–31			
Mean ± SD	24 ± 2.45			
Gender, n(%)				
Male	24	77.40%		
Female	7	22.60%		
Handedness, n(%)				
Left	0			
Right	31	100%		
Education level, n(%)				
Bachelor’s	21	67.70%		
Master’s or above	10	32.30%		
MOCA				
G1(M ± SD)	29.68 ± 0.60		3.13	0.05*
G2(M ± SD)	29.35 ± 1.14			
G3(M ± SD)	29.84 ± 0.37			
MMSE				
G1(M ± SD)	29.58 ± 0.81		1.71	0.19
G2(M ± SD)	29.55 ± 0.77			
G3(M ± SD)	29.84 ± 0.37			

M, Mean; SD, Standard Deviation; G1, Before simulated rapid high-altitude exposure; G2, After simulated rapid high-altitude exposure; G3, After TMS treatment; MOCA, Montreal Cognitive Assessment; MMSE, Mini-Mental State Examination. Significance levels are indicated as follows: *p < 0.05, **p < 0.01, ***p < 0.001.

### 3.2 Behavioral results

#### 3.2.1 Accuracy

**FIGURE 6:**
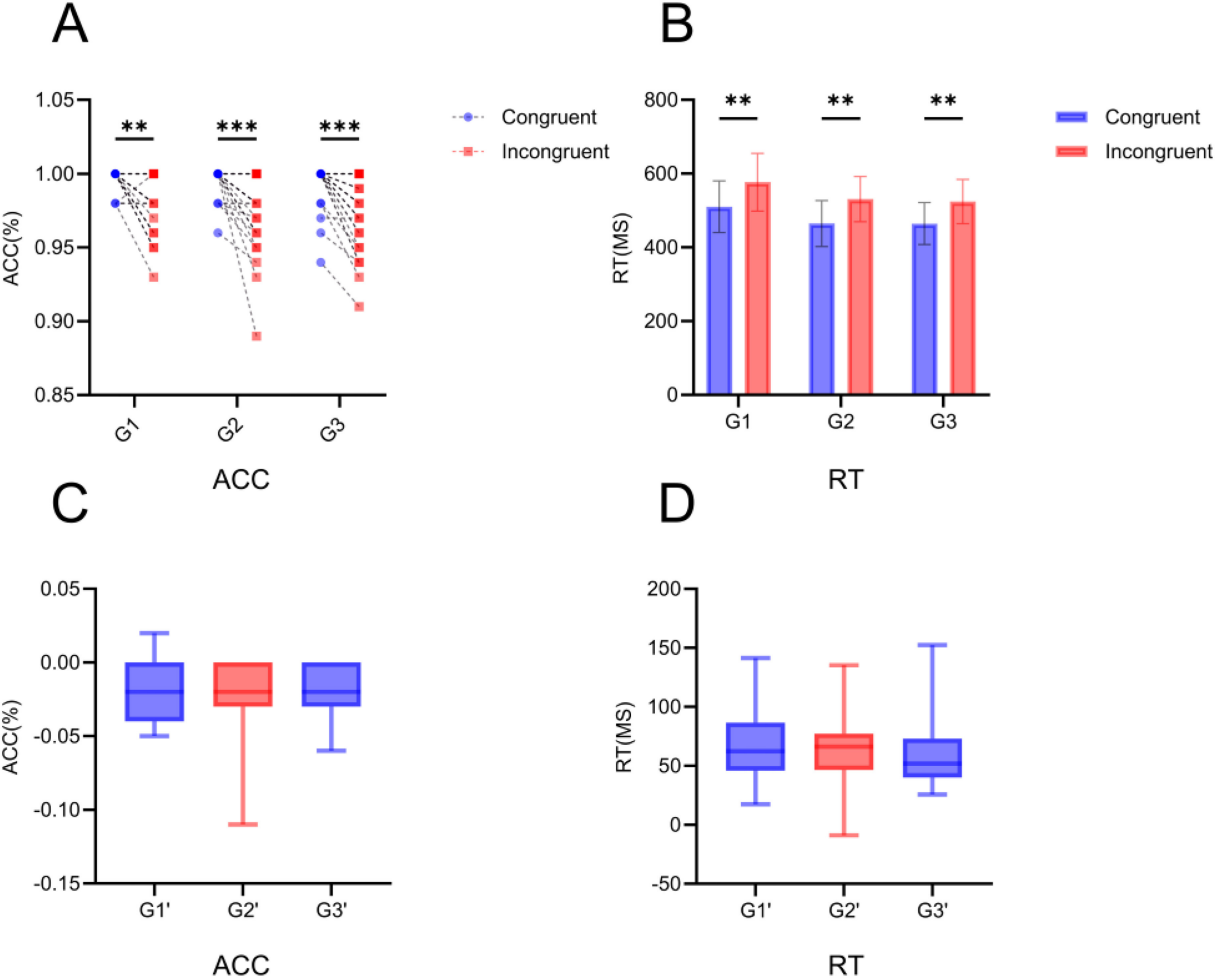
Behavioral results across time points and task conditions. **(A)** Mean accuracy (%) with individual scatter points. **(B)** Mean reaction time (ms). **(C)** Accuracy conflict effect size (incongruent - congruent). **(D)** RT conflict effect size (incongruent - congruent). Congruent: congruent condition; Incongruent: incongruent condition. G1: baseline; G2: post-24 h hypoxia; G3: post-rTMS. G1′–G3′: time points for conflict effect sizes. **p < 0.01, ***p < 0.001.

A repeated-measures ANOVA conducted on the accuracy of the Flanker task revealed a significant main effect of condition [*F*(1, 30) = 55.4, *p* < 0.001, η^2^ = 0.48], with accuracy for incongruent trials (mean 97.7%) being significantly lower than that for congruent trials (mean 99.6%). Neither the main effect of time [*F*(2, 60) = 1.4, *p* = 0.25, η^2^ = 0.05] nor the time × condition interaction [*F*(2, 60) = 0.3, *p* = 0.72, η^2^ = 0.01] reached statistical significance (Figures 6A,C and Table 2). These results demonstrate that although accuracy remained stable over time, the classical Flanker effect was consistently observed, with incongruent trials exhibiting lower accuracy across all three measurement occasions. The accuracy conflict effect size (incongruent − congruent) is shown across time points G1′, G2′, and G3′ in Figure 6C. Full statistical details are provided in Supplementary Table S1.

**TABLE 2:** Behavioral performance (RT and accuracy) across time points and conditions (M ± SD).

Condition	Measure	G1 (M ± SD)	G2 (M ± SD)	G3 (M ± SD)
Congruent	RT (ms)	510.5 ± 70.2	464.8 ± 62.3	464.5 ± 56.9
	Accuracy (%)	99.7 ± 0.7	99.5 ± 1.0	99.5 ± 1.4
Incongruent	RT (ms)	576.9 ± 78.3	531.2 ± 61.3	524.3 ± 60.0
	Accuracy (%)	98.0 ± 1.9	97.6 ± 2.6	97.4 ± 2.5

Values are M ± SD. ANOVA results are described in the text (for full statistical details, see Supplementary Table S1).

#### 3.2.2 Reaction time

The analysis of RT revealed a significant main effect of condition [*F*(1, 30) = 203.3, *p* < 0.001, η^2^ = 0.77], with longer RTs for incongruent trials (mean 544.2 ms) compared to congruent trials (mean 479.9 ms). A significant main effect of time was also observed [*F*(2, 60) = 23.2, *p* < 0.001, η^2^ = 0.44], while the time × condition interaction was not significant [*F*(2, 60) = 1.5, *p* = 0.24, η^2^ = 0.05] (Figures 6B,D and Table 2).

*Post-hoc* comparisons (Bonferroni-corrected) concerning the main effect of time revealed that RTs at G1 (mean 543.7 ms) were significantly longer than those at G2 (mean 498.0 ms, *p* < 0.001) and G3 (mean 494.4 ms, *p* < 0.001), with no significant difference between G2 and G3 (*p* = 0.43). When examining the effects of time across conditions, it was found that for incongruent trials, RTs at G1 (576.9 ms) were significantly longer than at G2 (531.2 ms, *p* < 0.001) and G3 (524.3 ms, *p* < 0.001). Similarly, for congruent trials, RTs at G1 (510.5 ms) were significantly longer than at G2 (464.8 ms, *p* < 0.001) and G3 (464.5 ms, *p* < 0.001). These findings suggest that RTs decreased significantly following interventions such as high-altitude exposure and rTMS when compared to baseline. The RT conflict effect size (incongruent − congruent) is shown across time points G1′, G2′, and G3′ in Figure 6D. Full statistical details are provided in Supplementary Table S1.

### 3.3 ERP results

#### 3.3.1 Scalp topography distribution

Figure 4 displays the scalp topographical distributions of the N2 and P3 components across different time points and task conditions. The N2 component demonstrated maximal amplitude predominantly in the frontal and frontocentral regions, while the P3 component was most prominent over the parietal and parieto-occipital regions. Analysis of the topographical maps across time points indicated a progressive increase in the negative amplitude of the N2 component from G1 to G3, accompanied by a corresponding increase in the positive amplitude of the P3 component. Furthermore, amplitudes elicited by incongruent trials were consistently larger than those elicited by congruent trials across all time points.

#### 3.3.2 P3 Component

Significant main effects of time on P3 amplitudes were observed at all four parietal electrodes [*F*(2, 60) = 3.8–15.9, *p* = 0.03 to < 0.001, η^2^ = 0.11–0.35]. *Post-hoc* comparisons revealed a progressive increase across time points: amplitudes at G3 were significantly higher than at G2 (all *p* < 0.05) and G1 (all *p* < 0.001), and amplitudes at G2 were significantly higher than at G1 (all *p* < 0.05). These findings suggest that both high-altitude exposure and subsequent rTMS significantly enhanced P3-related neural activity.

**FIGURE 7:**
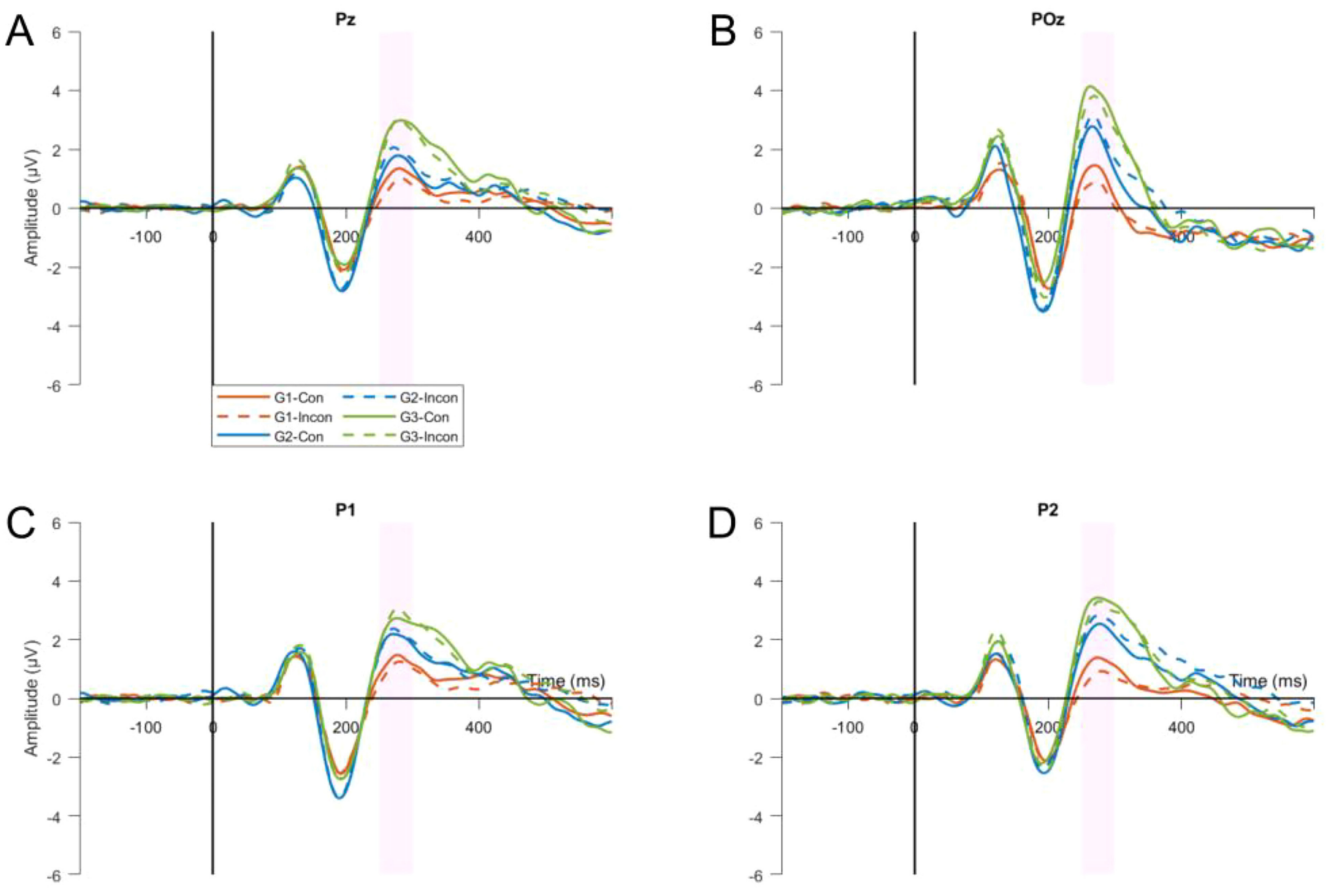
Grand-averaged P3 waveforms at parietal electrodes across time points and task conditions. (**A–D**) P3 waveforms at Pz, POz, P1, and P2 for congruent (Con) and incongruent (Incon) conditions at G1, G2, G3. Shaded pink areas indicate the P3 time window (250–300 ms).

**FIGURE 8:**
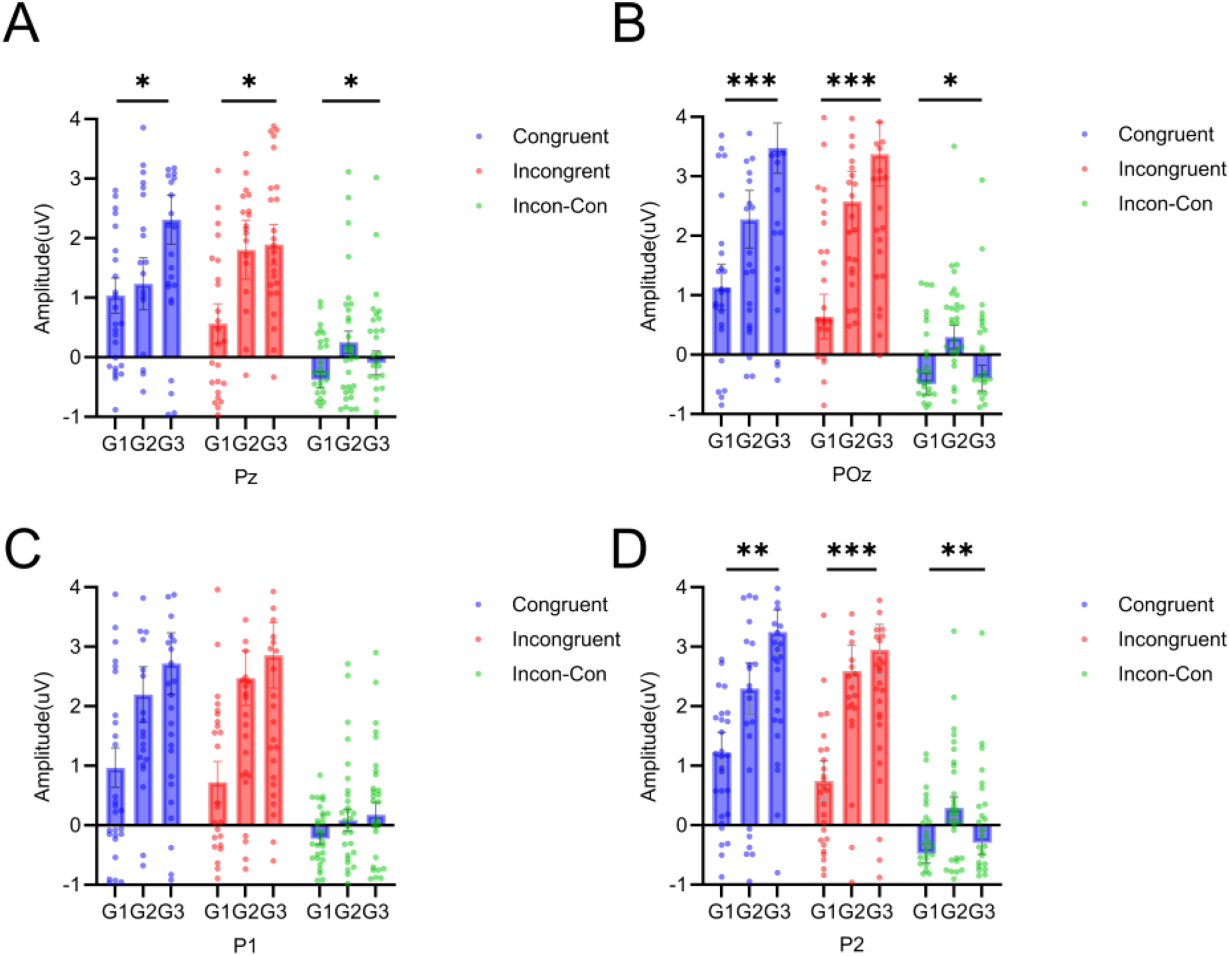
Mean P3 amplitudes at parietal electrodes across time points and task conditions. (**A–D**) Mean P3 amplitudes at Pz, POz, P1, and P2 electrodes for congruent (blue), incongruent (red), and conflict effect (Incon Con, green) conditions at three time points (G1, G2, G3). Individual participant data are overlaid as scatter points. *p < 0.05, **p < 0.01, ***p < 0.001.

No significant main effects of condition were observed at any electrode (all *p* > 0.05), indicating that P3 amplitudes did not significantly differ between incongruent and congruent trials when analyzed across all time points. The time × condition interaction was significant at POz [*F*(2, 60) = 5.4, *p* = 0.007, η^2^ = 0.15] and P2 [*F*(2, 60) = 4.7, *p* = 0.013, η^2^ = 0.13], and also reached significance at Pz [*F*(2, 60) = 3.3, *p* = 0.04, η^2^ = 0.10], but not at P1 (*p* > 0.05). Simple effect analyses indicated that the difference between incongruent and congruent trials (ΔP3) was relatively modest at G1 and G2 but increased at G3, particularly at POz and P2. For the conflict effect difference scores (ΔP3 = P3incongruent – P3congruent), one-way repeated-measures ANOVAs revealed a significant main effect of time at Pz, POz, and P2 [*F*(2, 60) = 3.3–5.4, all *p* < 0.05]. *Post hoc* tests showed that ΔP3 increased transiently from G1 to G2 (*p* < 0.01) and then returned to baseline at G3 (*p* > 0.05 vs. G1). No significant time effect was observed at P1 (*p* > 0.05). This pattern suggests that although high-altitude exposure transiently enhanced the allocation of attentional resources to conflict, the effect was not sustained, and rTMS did not further improve this specific efficiency. Detailed statistics for each electrode are provided in Table 3 (raw amplitudes) and Table 4 (difference scores), and the interaction is illustrated in Figures 7, 8. Detailed raw amplitude values and full statistical results are presented in Supplementary Table S2.

**TABLE 3:** Summary of ERP amplitude effects across time points and task conditions.

Component	Electrode	Time (p)	Condition (p)	Interaction (p)
N2	Fz	< 0.001	0.96	0.24
	FCz	0.01	0.15	0.49
	FC1	< 0.001	0.999	0.53
	FC2	0.04	0.95	0.26
	F1	< 0.001	0.43	0.32
	F2	< 0.001	0.88	0.24
P3	Pz	0.002	0.50	0.04
	POz	< 0.001	0.13	0.01
	P1	0.03	0.91	0.19
	P2	< 0.001	0.11	0.01

Results are from two-way repeated-measures ANOVA. Degrees of freedom: *df* = (2, 60) for time and time × condition interaction effects; *df* = (1, 30) for condition effect. *p*-values < 0.05 are bolded. Detailed mean amplitudes, *F*-values, and *post-hoc* comparisons are provided in Supplementary Table S2.

**TABLE 4:** Statistical results of conflict effects (ΔN2/ΔP3) for N2 and P3 components at different time points.

Component	Electrode	G1 Difference (M ± SD) (μV)	G2 Difference (M ± SD) (μV)	G3 Difference (M ± SD) (μV)	Main effect of Time (F, p, η^2^)	G1 vs. G2 (t, p)	G2 vs. G3 (t, p)	G1 vs. G3 (t, p)
N2	Fz	0.14 ± 0.99	−0.25 ± 1.50	0.08 ± 0.86	*F*(2, 60) = 1.48, *p* = 0.235, η^2^ = 0.047	*t*(30) = 1.45, *p* = 0.158	*t*(30) = −1.31, *p* = 0.201	*t*(30) = 0.30, *p* = 0.769
N2	FCz	−0.07 ± 0.98	−0.33 ± 1.12	−0.10 ± 0.89	*F*(2, 60) = 0.72, *p* = 0.493, η^2^ = 0.023	*t*(30) = 1.01, *p* = 0.319	*t*(30) = −1.11, *p* = 0.277	*t*(30) = 0.14, *p* = 0.893
N2	FC1	0.12 ± 0.89	0.03 ± 1.11	−0.15 ± 1.04	*F*(2, 60) = 0.64, *p* = 0.533, η^2^ = 0.021	*t*(30) = 0.35, *p* = 0.729	*t*(30) = 0.80, *p* = 0.428	*t*(30) = 1.11, *p* = 0.277
N2	FC2	0.15 ± 0.84	−0.19 ± 0.80	0.03 ± 1.07	*F*(2, 60) = 1.38, *p* = 0.260, η^2^ = 0.044	*t*(30) = 1.71, *p* = 0.098	*t*(30) = −1.17, *p* = 0.252	*t*(30) = 0.51, *p* = 0.613
N2	F1	0.27 ± 0.94	−0.11 ± 1.32	0.19 ± 1.25	*F*(2, 60) = 1.15, *p* = 0.324, η^2^ = 0.037	*t*(30) = 1.49, *p* = 0.147	*t*(30) = −1.07, *p* = 0.294	*t*(30) = 0.32, *p* = 0.749
N2	F2	0.11 ± 0.84	−0.19 ± 1.05	0.14 ± 1.03	*F*(2, 60) = 1.45, *p* = 0.242, η^2^ = 0.046	*t*(30) = 1.39, *p* = 0.176	*t*(30) = −1.55, *p* = 0.131	*t*(30) = −0.13, *p* = 0.896
P3	Pz	−0.38 ± 0.76	0.25 ± 1.04	−0.09 ± 1.11	*F*(2, 60) = 3.32, *p* = 0.043, η^2^ = 0.100	*t*(30) = −3.25, *p* = 0.003	*t*(30) = 1.17, *p* = 0.253	*t*(30) = −1.22, *p* = 0.231
P3	POz	−0.50 ± 1.00	0.30 ± 1.09	−0.39 ± 1.21	*F*(2, 60) = 5.35, *p* = 0.007, η^2^ = 0.151	*t*(30) = −3.90, *p* < 0.001	*t*(30) = 2.14, *p* = 0.040	*t*(30) = −0.40, *p* = 0.688
P3	P1	−0.23 ± 0.53	0.08 ± 1.01	0.18 ± 1.13	*F*(2, 60) = 1.72, *p* = 0.188, η^2^ = 0.054	*t*(30) = −1.79, *p* = 0.083	*t*(30) = −0.33, *p* = 0.746	*t*(30) = −1.87, *p* = 0.071
P3	P2	−0.48 ± 0.87	0.29 ± 1.02	−0.29 ± 1.10	*F*(2, 60) = 4.65, *p* = 0.013, η^2^ = 0.134	*t*(30) = −3.47, *p* = 0.002	*t*(30) = 1.96, *p* = 0.059	*t*(30) = −0.70, *p* = 0.488

ΔN2 = N2incongruent−N2congruent; ΔP3 = P3incongruent−P3congruent, representing the conflict effect size during conflict monitoring and attentional resource allocation stages, respectively. These values quantify the additional neural resources recruited by the brain to cope with conflict situations (neural efficiency). Data are expressed as mean ± standard deviation (M ± SD) in microvolts (μV). G1: Baseline (before hypobaric hypoxia exposure); G2: Post-exposure; G3: Post-rTMS intervention. Separate one-way repeated-measures ANOVAs were conducted for each electrode. Greenhouse–Geisser correction was applied when sphericity was violated. *Post-hoc* pairwise comparisons were performed using paired *t*-tests, and significance levels were adjusted using the Bonferroni method.

#### 3.3.3 N2 Component

Significant main effects of time on N2 amplitudes were observed at all six frontocentral electrodes [*F*(2, 60) = 3.4–10.6, *p* = 0.04 to < 0.001, η^2^ = 0.10–0.26]. *Post-hoc* comparisons indicated that N2 amplitudes (more negative) at both G2 and G3 were significantly larger than at G1 (all *p* < 0.01), with no significant difference between G2 and G3 (all *p* > 0.05). This pattern suggests that exposure to high altitude significantly enhanced conflict monitoring-related neural activity, which persisted after rTMS intervention without further intensification.

**FIGURE 9:**
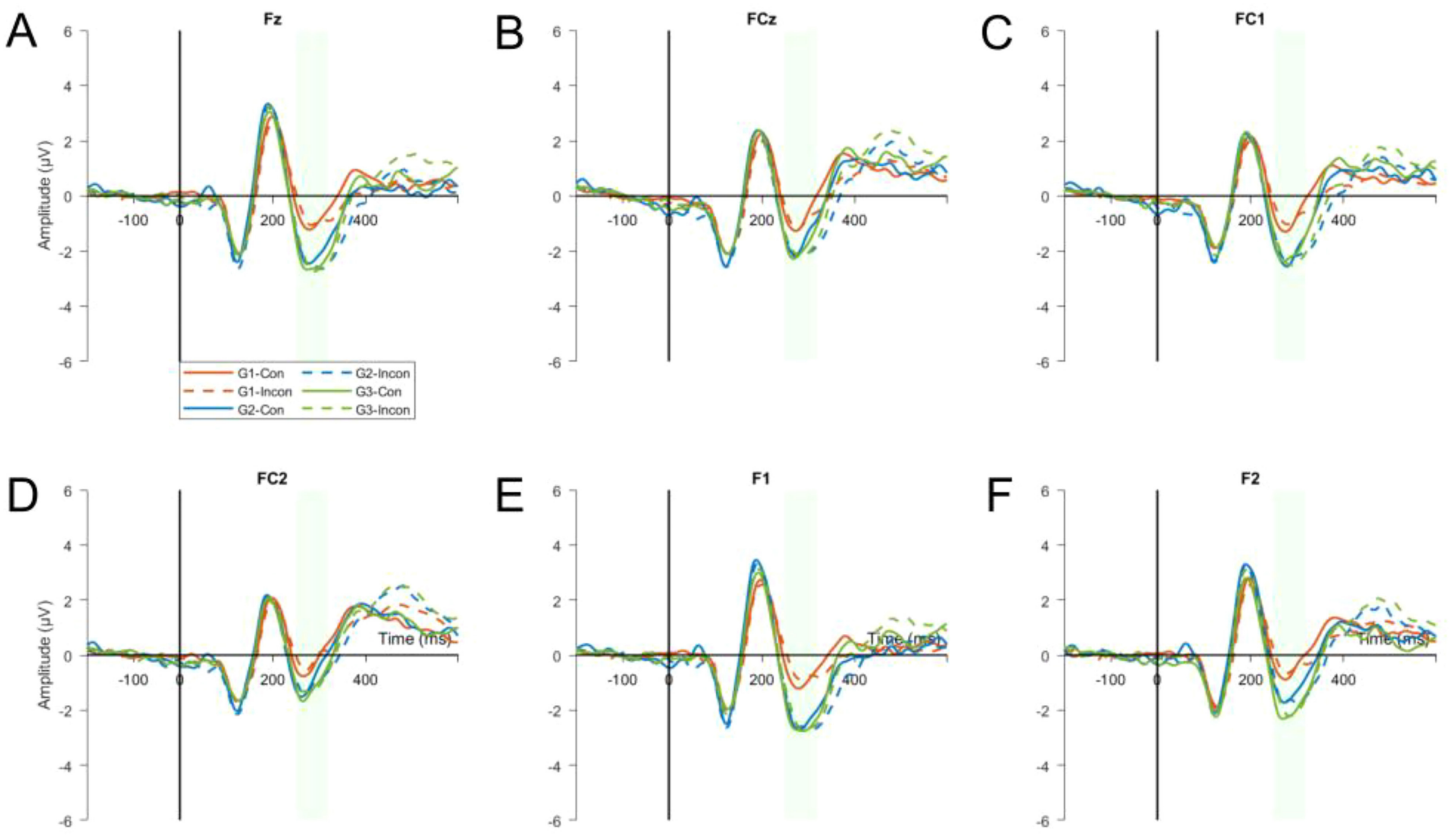
Grand-averaged N2 waveforms at frontocentral electrodes across time points and task conditions. (**A–F**) N2 waveforms at Fz, FCz, FC1, FC2, F1, and F2 for congruent (Con) and incongruent (Incon) conditions at G1, G2, G3. Shaded green areas indicate the N2 time window (250-320 ms).

**FIGURE 10:**
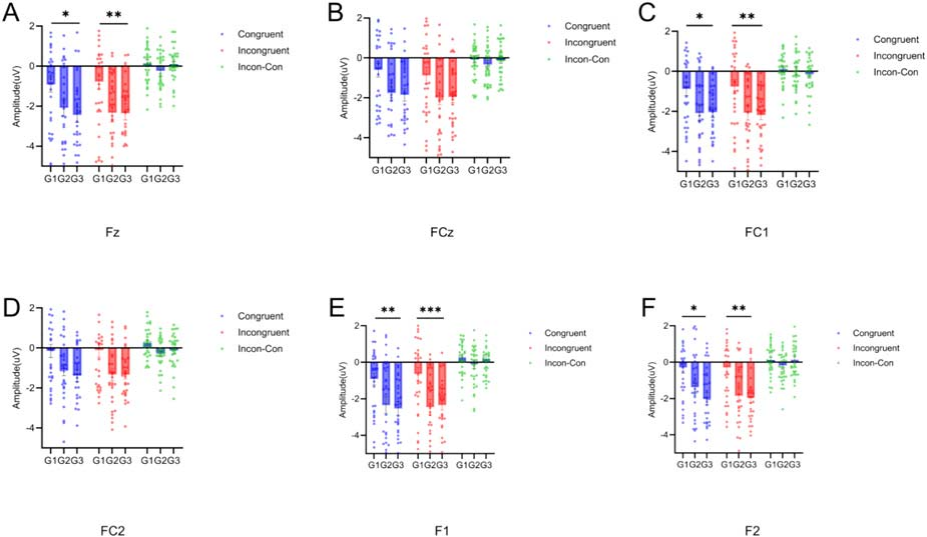
Mean N2 amplitudes at frontocentral electrodes across time points and task conditions. **(A–F)** Mean N2 amplitudes at Fz, FCz, FC1, FC2, F1, and F2 for congruent (blue), incongruent (red), and conflict effect (Incon Con, green) conditions at three time points (G1, G2, G3). Individual participant data are overlaid as scatter points. *p < 0.05, **p < 0.01, ***p < 0.001.

The main effects of condition were not significant at any electrode (all *p* > 0.05), indicating that N2 amplitudes were not significantly modulated by Flanker task congruency across time points. The time × condition interaction reached significance only at electrode F2 [*F*(2, 60) = 3.4, *p* = 0.04, η^2^ = 0.10] but not at the other five electrodes (all *p* > 0.05). Given that electrode-level analyses were exploratory and uncorrected for multiple comparisons, this single significant result is interpreted cautiously and not considered a robust effect. Simple effect analyses at F2 suggested a trend for a larger incongruent-minus-congruent difference at G2 and G3 compared to G1. However, direct analysis of the conflict effect difference scores (ΔN2 = N2incongruent – N2congruent) revealed no significant main effect of time at any electrode [*F*(2, 60) = 0.6–1.5, all *p* > 0.05; see Figures 9, 10 and Table 4]. This further confirms that the marginal interaction at F2 does not reflect a consistent or reliable modulation of conflict monitoring, reinforcing that conflict-related neural activity remained stable across time points. No significant main effects of time or condition were observed for N2 latency at any electrode (all *p* > 0.05). Detailed statistics for each electrode are provided in Table 3. Detailed raw amplitude values and full statistical results are presented in Supplementary Table S2.

#### 3.3.4 Conflict effect size analysis

We conducted one-way repeated-measures ANOVAs with time point (G1, G2, G3) as the within-subject factor on ΔN2 and ΔP3 values. For ΔN2, no significant main effect of time was observed at any electrode site: Fz [*F(*2, 60) = 1.5, *p* = 0.24, η^2^ = 0.05], FCz [*F*(2, 60) = 0.7, *p* = 0.49, η^2^ = 0.02], FC1 [*F*(2, 60) = 0.6, *p* = 0.53, η^2^ = 0.02], FC2 [*F*(2, 60) = 1.4, *p* = 0.26, η^2^ = 0.04], F1 [*F*(2, 60) = 1.2, *p* = 0.32, η^2^ = 0.04], and F2 [*F*(2, 60) = 1.5, *p* = 0.24, η^2^ = 0.05]. For ΔP3, a significant main effect of time was observed at Pz [*F*(2, 60) = 3.3, *p* = 0.04, η^2^ = 0.10], POz [*F*(2, 60) = 5.4, *p* = 0.007, η^2^ = 0.15], and P2 [*F*(2, 60) = 4.7, *p* = 0.013, η^2^ = 0.13], but not at P1 [*F*(2, 60) = 1.7, *p* = 0.19, η^2^ = 0.05]. Subsequent *post-hoc* paired *t*-tests indicated that ΔP3 at G2 was significantly greater than at G1 at Pz [*t*(30) = −3.3, *p* = 0.003], POz [*t*(30) = −3.9, *p* < 0.001], and P2 [*t*(30) = −3.5, *p* = 0.002]. This increase at G2 was followed by a significant decrease from G2 to G3 at POz [*t*(30) = 2.1, *p* = 0.04] and a marginal decrease at P2 [*t*(30) = 2.0, *p* = 0.06], culminating in no significant differences between G3 and G1 at any electrode (all *p* > 0.05). These findings are summarized in Table 4.

These results indicate that while the raw N2 and P3 amplitudes were significantly elevated at G2 and G3 (as shown in Table 3), the conflict effect difference scores provide a more detailed perspective. Specifically, ΔN2 did not exhibit significant differences across the time points, and although ΔP3 demonstrated fluctuations, it ultimately reverted to baseline levels at G3, suggesting no sustained enhancement in processing efficiency.

## 4 Discussion

The current study utilized a repeated-measures design that integrated a 24-h simulated high-altitude (3,600 meters) hypobaric hypoxia exposure with task-state EEG and rTMS interventions to investigate the effects of acute high-altitude stress on conflict monitoring and cognitive control functions, as well as the exploratory modulatory impact of rTMS. Four principal findings were observed.

First, at the behavioral level, RTs significantly decreased following high-altitude exposure and rTMS intervention. This result, which contrasts with some previous studies reporting hypoxia-induced prolongation of RTs (Bliemsrieder et al., 2022), suggests that practice effects and possibly increased arousal could have influenced behavioral performance. This is reflected in a speed-accuracy trade-off, as evidenced by a slight, non-significant decline in accuracy. Second, at the neurophysiological level, N2 amplitudes became significantly more negative (i.e., larger in magnitude) and P3 amplitudes became significantly more positive after high-altitude exposure. Although the overall main effects of congruency were not significant, significant time-by-condition interactions were observed at select electrodes (P2, POz for P3; F2 for N2). Given that electrode-level analyses were exploratory and uncorrected for multiple comparisons, these results are interpreted descriptively and do not indicate robust, global modulation of conflict processing by the interventions. This finding suggests enhanced neural activity related to conflict monitoring and attentional resource allocation, with a specific amplification of conflict-related processing following rTMS. Third, P3 amplitudes became even more positive following rTMS intervention, and the amplitude difference between incongruent and congruent conditions at G3 was more pronounced compared to G1 and G2, suggesting that rTMS may be associated with enhanced neural responses to conflict stimuli (exploratory). Fourthly, and most critically, despite the enhancement of raw neural activity, conflict effect difference scores (ΔN2, ΔP3), which isolate neural activity specifically engaged in conflict processing, showed no significant differences across time points. This pattern of “increased neural activity without selective enhancement in conflict processing” provides direct electrophysiological evidence that neural recruitment under acute high-altitude stress is non-specific.

### 4.1 Behavioral performance: learning effects and speed-accuracy trade-off

Behavioral results indicated that RTs on the Flanker task were significantly shorter at G2 (immediately post-exposure) and G3 (post-rTMS) compared to G1 (baseline), while accuracy exhibited a slight, albeit non-significant, decline. This finding contrasts with some previous studies that reported prolonged RTs following acute high-altitude exposure (Bliemsrieder et al., 2022). The most plausible explanation for this discrepancy involves learning effects (Liu and Reder, 2016). The repeated task administration across three time points introduces unavoidable practice effects, which may independently contribute to faster response speeds and greater task automation. Although the Flanker task requires conflict monitoring, repeated exposure might facilitate automatic processing of stimulus-response mappings, consequently reducing RTs.

Additionally, the arousing effects of acute mild hypoxia may have contributed to these findings. During the initial stages of acute hypoxia, activation of the sympathetic nervous system increases catecholamine release, potentially elevating alertness and accelerating response speeds (Zhong et al., 2021). This physiological response may partially counteract the cognitive impairments typically associated with early hypoxic exposure. However, the slight decrease in accuracy suggests an adjustment in the speed-accuracy trade-off. Participants may have sacrificed some accuracy to maintain faster response speeds, representing an adaptive strategy under hypoxic conditions where rapid responses may be prioritized over perfect accuracy.

### 4.2 N2 component: compensatory enhancement and selectivity dilemma in conflict monitoring

The N2 component, closely associated with conflict monitoring and primarily emanating from the ACC (Larson et al., 2014; Hong et al., 2021), exhibited significantly larger (more negative) amplitudes at all electrode sites during phases G2 and G3 compared to G1. This suggests that 24-h high-altitude exposure substantially enhances conflict monitoring-related neural activity, an effect that persists following exploratory rTMS intervention. From a neurophysiological standpoint, this may reflect hypoxia-induced neuroplastic changes. The ACC is highly sensitive to hypoxia; acute exposure may activate ACC neurons and enhance local field potential oscillations, manifesting as increased N2 amplitudes (Liu et al., 2025; Taylor et al., 2025). Concurrently, this phenomenon may represent compensatory cognitive resource recruitment, with the brain mobilizing additional neural resources to maintain cognitive function while lacking specificity in conflict-related processing.

However, the analysis of the main effects for each condition revealed that, contrary to the typical Flanker effect, N2 amplitudes were not significantly modulated by congruency when aggregated across all time points (all *p*-values > 0.05, see Table 3). This unexpected result suggests that although high-altitude exposure increased the overall intensity of neural activity (as indicated by N2 amplitude), it did not enhance the brain’s ability to differentially process congruent versus incongruent stimuli at the level of conflict monitoring. The fundamental capability for conflict detection, as reflected by the N2 difference wave, remained stable and was not significantly amplified by either hypoxia or rTMS. A significant time-by-condition interaction was observed only at F2, with a trend at F1. Given the lack of multiple-comparison correction and the single-electrode nature of this effect, it does not provide reliable evidence for altered conflict sensitivity and is interpreted cautiously.

Crucially, a one-way ANOVA on conflict effect sizes (ΔN2) revealed no significant differences across time points at any electrode site. This finding constitutes the core observation: although raw N2 amplitudes increased significantly, the additional neural resources specifically recruited for conflict processing did not exhibit corresponding increases. In other words, while the brain indeed invested more in conflict monitoring following high-altitude exposure, these additional investments did not translate into enhanced conflict-specific processing. This reflects a pattern of non-specific or excessive neural recruitment, where increased resource recruitment does not yield finer conflict resolution.

The absence of significant changes in ΔN2 also challenges alternative explanations that amplitude increases merely reflect heightened general alertness or task difficulty. If amplitude increases resulted from general factors, the amplitudes for both congruent and incongruent conditions would increase proportionally, leaving ΔN2 unchanged or reduced. The observed stability of ΔN2 thus supports interpretations of “compensatory recruitment” rather than “general enhancement.”

### 4.3 P3 component: cumulative enhancement and selectivity bottleneck in attentional resource allocation

The P3 component, associated with context updating, decision closure, and attentional resource allocation, primarily involves the parietal regions, temporoparietal junctions, and posterior cingulate cortex (Volpe et al., 2007). As shown in Table 3, the baseline (G1) P3 amplitudes at parietal electrodes ranged from approximately 0.6–1.3 μV, which is within the typical physiological range. Analysis of the main effects over time revealed a graded increase in P3 amplitudes across all electrode sites: G3 > G2 > G1. This observation yields two critical insights. Firstly, exposure to high altitude for 24 h alone significantly enhanced P3 amplitudes (G2 > G1), indicating the mobilization of attentional resources under stress. Secondly, three consecutive days of exploratory rTMS intervention further amplified this enhancement (G3 > G2), demonstrating potential cumulative neuromodulatory effects.

Interestingly, the effects of the condition across parietal electrodes were not significant, as evidenced by *p* > 0.05 (refer to Table 3). This finding indicates that P3 amplitudes did not vary significantly between incongruent and congruent trials. Nonetheless, a significant interaction between time and condition was detected at the P2 and POz electrodes. Analysis of simple effects showed that the difference in P3 amplitudes between incongruent and congruent conditions (ΔP3) was negligible at G1 and G2 but became markedly more pronounced at G3, especially at POz. This result suggests that rTMS may be associated with enhanced neural responses to conflict stimuli (exploratory).

Further analysis of the conflict effect sizes (ΔP3) revealed a more complex pattern than merely a lack of effect. A significant main effect of time was identified, characterized by an initial increase in ΔP3 from G1 to G2, followed by a decrease back to G1 levels at G3 (refer to Table 4). This pattern suggests that exposure to high altitude temporarily enhanced the allocation of attentional resources toward conflict at G2; however, this enhancement was not maintained, as the effect returned to baseline levels following rTMS intervention. In the absence of a sham-rTMS control, we cannot definitively conclude that rTMS failed to augment conflict-specific attentional allocation. The observed return of ΔP3 to baseline at G3 may reflect habituation, regression to the mean, or spontaneous recovery, rather than the absence of an rTMS effect. Consequently, although there was a significant increase in raw P3 amplitudes at G2 and G3, and rTMS further amplified this increase, the allocation of additional attentional resources for conflict processing exhibited only a transient enhancement at G2 that was not sustained, returning to baseline at G3. This pattern of results indicates that the resource enhancement observed following rTMS was broad and non-selective, with the brain mobilizing resources generally rather than directing them specifically for conflict processing.

### 4.4 Integrated analysis of conflict effect sizes: neural-behavioral dissociation and non-specific neural recruitment

The conflict effect difference score approach (incongruent minus congruent) has been validated as a sensitive method to quantify task-specific neural activation, as demonstrated in recent ERP and EEG-fMRI studies (Ebrahimzadeh et al., 2025a; Mohammadi et al., 2025). An analysis synthesizing conflict effect sizes for N2 and P3 components has uncovered a significant finding: exposure to high altitudes notably increases the raw amplitudes of N2 and P3, suggesting intensified neural activity associated with conflict monitoring and attentional resource allocation. Although this indicates an enhancement in overall neural activity, the specificity of conflict processing, measured by the conflict effect sizes, is unchanged. Specifically, ΔN2 remained constant across all time points, signifying no alteration in conflict monitoring specificity. Conversely, ΔP3 exhibited a transient increase immediately following high-altitude exposure (G2) but reverted to baseline levels after exploratory rTMS intervention (G3), indicating no enduring improvement in the specificity of attentional allocation. This pattern of “neural enhancement without improved selectivity” provides direct electrophysiological support for non-specific neural recruitment under acute high-altitude stress.

This suggests that the increased neural activity does not translate into finer higher-level conflict processing. Concurrently, behavioral performance demonstrates shortened RTs alongside a slight decrease in accuracy, indicative of adjustments based on a speed-accuracy trade-off.

This pattern of “neural enhancement without improved specificity” exemplifies a classic neural-behavioral dissociation phenomenon, providing direct electrophysiological evidence for non-specific neural recruitment under stress. Non-specific neural recruitment refers to the brain’s recruitment of additional neural resources to sustain fundamental cognitive functions in response to endogenous or exogenous challenges, such as hypoxia. However, this mobilization is non-selective because increased investment does not yield proportional improvements in task-specific performance.

From the perspective of energy metabolism, non-specific recruitment might reflect imbalances between the cerebral energy supply and demand under hypoxic conditions (Ortiz-Prado et al., 2025). As the most oxygen-consuming organ in the body, the brain is particularly sensitive to hypoxia. Under such conditions, the efficacy of neuronal oxidative phosphorylation declines, ATP production diminishes, and neural networks must compensate with higher discharge rates to maintain cognitive function. This results in elevated neural energy consumption per unit of cognitive output.

From the perspective of neural transmission, hypoxia may impair the synthesis and release of monoamine neurotransmitters, such as dopamine and norepinephrine, thereby reducing the quality and effectiveness of neural signal transmission (Zhong et al., 2021). Animal studies have shown that hypoxic exposure significantly alters the turnover rates of dopamine and norepinephrine in cortical and subcortical regions, potentially contributing to the observed dissociation between enhanced neural activity and stable behavioral performance.

From the perspective of neural network coordination, functional connectivity within the frontoparietal networks may reorganize under hypoxic conditions, with reduced synchrony, complicating the task of achieving effective functional integration at the global level through enhanced local neural activity.

### 4.5 Modulatory effects of rTMS intervention: enhancing neural activity without improving selectivity

The present study found that rTMS intervention significantly enhanced cumulative effects on P3 amplitudes; however, it did not increase N2 amplitudes beyond those induced by hypoxia, nor did it significantly affect ΔN2 or ΔP3. These results indicate both component specificity and limitations in the ability of rTMS to refine conflict-related neural activity. Previous studies have systematically evaluated rTMS parameters for DLPFC modulation, including frequency, intensity, duration, and hemisphere, providing benchmarks for cognitive enhancement protocols (Asgarinejad et al., 2024; Ebrahimzadeh et al., 2024; Ebrahimzadeh et al., 2025b).

Component specificity is evidenced by the stronger rTMS effects on P3 compared to N2, which may be associated with the functional properties of the stimulation target, the right DLPFC). The DLPFC is a central node in executive control networks, playing vital roles in the allocation of attention through its functional connections with the ACC and parietal regions (Cieslik et al., 2013). Stimulation of the DLPFC may enhance the top-down regulation of parietal attention networks, as reflected by increased P3 amplitudes. In contrast, N2 primarily reflects conflict monitoring functions mediated by the ACC, which has relatively weaker functional connections with the DLPFC.

As an exploratory observation without a sham control condition, rTMS was associated with elevated P3 amplitude, but no definitive conclusions can be drawn regarding its specific efficacy in regulating conflict-related neural activity. Despite the increase in P3 amplitude following rTMS, there was no corresponding improvement in conflict-specific processing, as indicated by the unchanged ΔP3. This dissociation underscores that an enhanced neural response magnitude does not necessarily translate into greater processing selectivity. Improving conflict-specific processing would likely require concurrent improvements in signal-to-noise discrimination, more precise neural encoding, and increased synchrony within task-relevant networks. Although the 1 Hz low-frequency rTMS protocol employed in this study was effective in modulating cortical excitability, its ability to improve higher-order network-level selectivity appears to be limited.

Future research should explore whether alternative stimulation parameters, such as higher frequencies (e.g., 10 Hz) or intermittent theta burst stimulation (iTBS), might more effectively enhance both the intensity and selectivity of neural activity. This could potentially be achieved by inducing different patterns of synaptic plasticity and network reorganization.

### 4.6 Novel contributions and theoretical implications

This study presents several significant contributions. First, it systematically validates the phenomenon of “neural enhancement without improved specificity” following exposure to high-altitude stress, thus offering insights into the neural mechanisms underlying cognitive impairments at high altitudes. Second, it introduces the “non-specific neural recruitment” framework, which expands upon the conventional view that “neural activity enhancement equals functional compensation” by emphasizing resource selectivity rather than general activation. Third, the study employs conflict effect sizes (incongruent minus congruent) as primary analytical indices. This methodological innovation distinguishes general stimulus-related neural activity from task-specific neural processing during conflict processing, a central cognitive process. Fourth, this research conducts the first exploration of rTMS modulation effects on cognitive functions after high-altitude exposure from a neuroelectrophysiological standpoint, thereby extending neuromodulation techniques to the field of environmental medicine.

### 4.7 Limitations and future directions

The study has several limitations that merit attention. First, the sample size (*N* = 31), while sufficient to detect main effects of time and condition, may not provide enough statistical power to detect higher-order interaction effects at specific electrode sites. Consequently, the interaction effects observed at electrodes F2 and P2 should be approached with caution and validated through further studies.

Second, the lack of a control group prevents definitive attribution of the observed changes solely to hypoxia or rTMS, as practice or learning effects could have contributed (e.g., faster RTs at G2 and G3). However, our core conclusions regarding conflict processing specificity are based on conflict effect difference scores (ΔN2, ΔP3; incongruent minus congruent). These difference scores isolate neural activity specific to conflict processing and are less susceptible to general practice effects than raw amplitudes. Any systematic learning effect would be expected to similarly reduce both congruent and incongruent trial amplitudes, leaving the difference scores largely unchanged. Thus, while practice may explain the overall RT speeding, it cannot account for the stable specificity of conflict processing. Nonetheless, future studies should include an active control group (e.g., a normoxic or sham-rTMS group) to disentangle treatment effects from time-related biases.

Third, the rTMS protocol was exploratory, utilizing a single set of stimulation parameters (1 Hz, 40% RMT, right DLPFC), which precludes comparisons across different frequencies, intensities, or stimulation protocols. Fourth, the investigation was limited to time-domain ERP analyses; it did not include time-frequency decompositions or assessments of functional connectivity, thus restricting deeper insights into oscillatory dynamics and network-level interactions. Fifth, the simulated high-altitude environment may not fully mimic actual high-altitude conditions, where elements such as low temperature and ultraviolet radiation may interact with hypoxic stress.

These limitations suggest several concrete and actionable avenues for future research. First, future studies should adopt sham-controlled, double-blind designs to clarify the causal effects of rTMS and distinguish treatment effects from practice or time effects. Second, validation studies should be conducted in real high-altitude environments (e.g., at an elevation of 3,600 m in Tibet or Nepal) using the same Flanker-ERP paradigm to improve ecological validity beyond simulated hypobaric hypoxia. Third, future work should examine whether conflict effect sizes (ΔN2, ΔP3) can serve as neurophysiological biomarkers for screening individuals susceptible to high-altitude cognitive impairment. Fourth, optimized rTMS protocols with varying frequencies, intensities, and targets should be compared to identify parameters that can improve conflict-specific processing rather than merely increasing overall neural activity. Fifth, longitudinal studies are needed to explore the persistence of neurocognitive changes and non-specific neural recruitment during longer-term high-altitude exposure.

## 5 Conclusion

The present study demonstrates that acute high-altitude stress enhances the intensity of neural activity related to conflict monitoring (N2) and attentional allocation (P3). However, this enhancement in overall neural activity does not correspond to selective improvement in conflict-specific processing, as indexed by the conflict effect sizes. Notably, ΔN2 remained unchanged across all time points, and ΔP3 showed only a transient increase immediately after exposure that was not sustained, returning to baseline levels following rTMS. This pattern of increased overall neural activity without proportional enhancement in conflict-selective processing supports the view that neural recruitment under hypoxic stress is non-specifically augmented. Exploratory findings regarding rTMS intervention showed that it further amplified P3 amplitude but did not selectively modulate conflict-related processing, suggesting a preliminary effect on overall neural response magnitude rather than targeted resource allocation. Conflict effect sizes (ΔN2, ΔP3) may serve as sensitive markers for assessing neurocognitive states and evaluating neuromodulatory interventions in environmental neuroscience.

## Data Availability

The original contributions presented in the study are included in the article/Supplementary material, further inquiries can be directed to the corresponding authors.

## References

[B1] Agbangla N. F. Audiffren M. Pylouster J. Albinet C. T. (2019). Working memory, cognitive load andcardiorespiratory fitness: testing the CRUNCHModel with near-infrared spectroscopy. *Brain Sci.* 9:38. 10.3390/brainsci9020038 30744137 PMC6406418

[B2] Asgarinejad M. Saviz M. Sadjadi S. M. Saliminia S. Kakaei A. Esmaeili P. et al. (2024). Repetitive transcranial magnetic stimulation (rTMS) as a tool for cognitive enhancement in healthy adults: a review study. *Med. Biol. Eng. Comput.* 62 653–673. 10.1007/s11517-023-02968-y 38044385

[B3] Bell A. J. Sejnowski T. J. (1995). An information-maximization approach to blind separation and blind deconvolution. *Neural Comput.* 7 1129–1159. 10.1162/neco.1995.7.6.1129 7584893

[B4] Bliemsrieder K. Weiss E. M. Fischer R. Brugger H. Sperner-Unterweger B. Hüfner K. et al. (2022). Cognition and neuropsychological changes at altitude-a systematic review of literature. *Brain Sci.* 12:1736. 10.3390/brainsci12121736 36552195 PMC9775937

[B5] Cambiaghi M. Crupi R. Bautista E. L. Elsamadisi A. Malik W. Pozdniakova H. et al. (2020). The effects of 1-Hz rTMS on emotional behavior and dendritic complexity of mature and newly generated dentate gyrus neurons in male mice. *Int. J. Environ. Res. Public Health* 17:4074. 10.3390/ijerph17114074 32521613 PMC7312937

[B6] Chen Q. Wang Z. J. Wang N. N. Su R. Yu S. F. Huang X. Y. et al. (2025). High-altitude adaptation strategies: active integration of higher-order cognitive processes by indigenous residents to overcome altitude-induced constraints in visual cognition. *Neuroscience* 581 142–156. 10.1016/j.neuroscience.2025.07.015 40645322

[B7] Chen X. Zhang Q. Wang J. Liu J. Zhang W. Qi S. et al. (2017). Cognitive and neuroimaging changes in healthy immigrants upon relocation to a high altitude: a panel study. *Hum. Brain Mapp*. 38 3865–3877. 10.1002/hbm.23635 28480993 PMC6867044

[B8] Cieslik E. C. Zilles K. Caspers S. Roski C. Kellermann T. S. Jakobs O. et al. (2013). Is there “one”; DLPFC in cognitive action control? Evidence for heterogeneity from co-activation-based parcellation. *Cereb. Cortex* 23 2677–2689. 10.1093/cercor/bhs256 22918987 PMC3792742

[B9] Clawson A. Clayson P. E. Keith C. M. Catron C. Larson M. J. (2017). Conflict and performance monitoring throughout the lifespan: an event-related potential (ERP) and temporospatial component analysis. *Biol. Psychol*. 124 87–99. 10.1016/j.biopsycho.2017.01.012 28143802

[B10] Clayson P. E. Larson M. J. (2011). Conflict adaptation and sequential trial effects: support for the conflict monitoring theory. *Neuropsychologia* 49 1953–1961. 10.1016/j.neuropsychologia.2011.03.023 21435347

[B11] Coronel-Oliveros C. Medel V. Whitaker G. A. Astudillo A. Gallagher D. Z-Rivera L. et al. (2024). Elevating understanding: linking high-altitude hypoxia to brain aging through EEG functional connectivity and spectral analyses. *Netw. Neurosci.* 8 275–292. 10.1162/netn_a_00352 38562297 PMC10927308

[B12] Duan K. Xie S. Zhang X. Xie X. Cui Y. Liu R. et al. (2023). Exploring the temporal patterns of dynamic information flow during attention network test (ANT). *Brain Sci*. 13:247. 10.3390/brainsci13020247 36831790 PMC9954291

[B13] Duncan C. C. Barry R. J. Connolly J. F. Fischer C. Michie P. T. Näätänen R. et al. (2009). Event-related potentials in clinical research: guidelines for eliciting, recording, and quantifying mismatch negativity, P300, and N400. *Clin. Neurophysiol*. 120 1883–1908. 10.1016/j.clinph.2009.07.045 19796989

[B14] Ebrahimzadeh E. Alavi S. M. Bijar A. Pakkhesal A. (2013). A novel approach for detection of deception using Smoothed Pseudo Wigner-Ville Distribution (SPWVD). *J. Biomed. Sci. Eng.* 6 8–18. 10.4236/jbise.2013.61002

[B15] Ebrahimzadeh E. Dehghani A. Asgarinejad M. Soltanian-Zadeh H. (2024). Non-linear processing and reinforcement learning to predict rTMS treatment response in depression. *Psychiatry Res.* 337:111764. 10.1016/j.pscychresns.2023.111764 38043370

[B16] Ebrahimzadeh E. Mohammadi A. M. Hammoud A. Rajabion L. (2025a). “Neural correlates of reward and punishment processing during gambling-based decision-making: a simultaneous EEG-fMRI Study,” in *Proceedings of the 2025 32nd National and 10th International Iranian Conference on Biomedical Engineering (ICBME)*, (Tabriz: IEEE).

[B17] Ebrahimzadeh E. Sadjadi S. M. Asgarinejad M. Dehghani A. Rajabion L. Soltanian-Zadeh H. (2025b). Neuroenhancement by repetitive transcranial magnetic stimulation (rTMS) on DLPFC in healthy adults. *Cogn. Neurodyn*. 19:34. 10.1007/s11571-024-10195-w 39866659 PMC11759757

[B18] Ebrahimzadeh E. Soltanian-Zadeh H. (2026). Cognitive enhancement by brain stimulation techniques. *Front. Media SA* 20:1824005. 10.3389/fnhum.2026.1824005 41993068 PMC13079620

[B19] Falla M. Papagno C. Dal Cappello T. Vögele A. Hüfner K. Kim J. et al. (2021). A prospective evaluation of the acute effects of high altitude on cognitive and physiological functions in lowlanders. *Front Physiol*. 12:670278. 10.3389/fphys.2021.670278 33995130 PMC8113692

[B20] Faul F. Erdfelder E. Lang A. G. Buchner A. (2007). G*Power 3: a flexible statistical power analysis program for the social, behavioral, and biomedical sciences. *Behav. Res. Methods* 39 175–191. 10.3758/bf03193146 17695343

[B21] Forster H. V. Soto R. J. Dempsey J. A. Hosko M. J. (1975). Effect of sojourn at 4,300 m altitude on electroencephalogram and visual evoked response. *J. Appl. Physiol*. 39 109–113. 10.1152/jappl.1975.39.1.109 1150576

[B22] Fuggetta G. Pavone E. F. Fiaschi A. Manganotti P. (2008). Acute modulation of cortical oscillatory activities during short trains of high-frequency repetitive transcranial magnetic stimulation of the human motor cortex: a combined EEG and TMS study. *Hum. Brain Mapp*. 29 1–13. 10.1002/hbm.20371 17318833 PMC6870897

[B23] Gong H. Liu Y. X. Xiaoluo Q. L. Gong M. F. Liu Z. Wu S. R. et al. (2025). Unraveling the complexity of cognitive impairment following high-altitude exposure: from preclinical animal models to human organoids. *Front. Neurosci*. 19:1679858. 10.3389/fnins.2025.1679858 41333067 PMC12665664

[B24] Hong X. Yang F. Wang J. Li C. Ding M. Sheng J. (2021). Conflict Processing in Schizophrenia: dissociable neural mechanisms revealed by the N2 and frontal midline theta. *Neuropsychologia* 155:107791. 10.1016/j.neuropsychologia.2021.107791 33610613

[B25] Jiang J. van Gaal S. Bailey K. Chen A. Zhang Q. (2013). Electrophysiological correlates of block-wise strategic adaptations to consciously and unconsciously triggered conflict. *Neuropsychologia* 51 2791–2798. 10.1016/j.neuropsychologia.2013.09.020 24055539

[B26] Kanske P. Kotz S. A. (2010). Modulation of early conflict processing: n200 responses to emotional words in a flanker task. *Neuropsychologia* 48 3661–3664. 10.1016/j.neuropsychologia.2010.07.021 20654636

[B27] King E. S. Tang A. D. (2024). Intrinsic plasticity mechanisms of repetitive transcranial magnetic stimulation]. *Neuroscientist* 30 260–274. 10.1177/10738584221118262 36059273 PMC10928958

[B28] Larson M. J. Clayson P. E. Clawson A. (2014). Making sense of all the conflict: a theoretical review and critique of conflict-related ERPs. *Int. J. Psychophysiol.* 93 283–297. 10.1016/j.ijpsycho.2014.06.007 24950132

[B29] Lefaucheur J. P. Aleman A. Baeken C. Benninger D. H. Brunelin J. Di Lazzaro V. et al. (2020). Evidence-based guidelines on the therapeutic use of repetitive transcranial magnetic stimulation (rTMS): an update (2014-2018). *Clin. Neurophysiol.* 131 474–528. 10.1016/j.clinph.2019.11.002 31901449

[B30] Liu X. Wang X. Zhuang X. Qiu S. Tang Y. Qin Y. (2025). Transcranial magnetic stimulation regulates brain functional network dynamics in stroke patients: a randomised controlled trial. *J. Neuroeng. Rehabil.* 22:235. 10.1186/s12984-025-01776-9 41214733 PMC12599113

[B31] Liu X. L. Reder L. M. (2016). Fmri exploration of pedagogical benefits of repeated testing: when more is not always better. *Brain Behav.* 6:e00476. 10.1002/brb3.476 27458542 PMC4875931

[B32] Liu Z. Yu S. Hu Y. Wang D. Wang S. Tang Z. et al. (2023). Efficacy and safety of repeated transcranial magnetic stimulation combined with escitalopram in the treatment of major depressive disorder: a meta-analysis. *Front. Psychiatry* 14:1275839. 10.3389/fpsyt.2023.1275839 38234362 PMC10791764

[B33] Mantovani A. Aly M. Dagan Y. Allart A. Lisanby S. H. (2013). Randomized sham controlled trial of repetitive transcranial magnetic stimulation to the dorsolateral prefrontal cortex for the treatment of panic disorder with comorbid major depression. *J. Affect. Disord.* 144 153–159. 10.1016/j.jad.2012.05.038 22858212

[B34] McGuire S. A. Ryan M. C. Sherman P. M. Sladky J. H. Rowland L. M. Wijtenburg S. A. et al. (2019). White matter and hypoxic hypobaria in humans. *Hum. Brain Mapp.* 40 3165–3173. 10.1002/hbm.24587 30927318 PMC6592734

[B35] Mohammadi A. M. Mahmoudi S. Amin N. Hosseinzadeh F. (2025). “Neural encoding of outcome magnitude: evidence from fMRI,” in *Proceedings of the 2025 32nd National and 10th International Iranian Conference on Biomedical Engineering (ICBME)*, (Tabriz: IEEE).

[B36] Moretti J. Rodger J. (2022). A little goes a long way: neurobiological effects of low intensity rTMS and implications for mechanisms of rTMS. *Curr. Res. Neurobiol.* 3:100033. 10.1016/j.crneur.2022.100033 36685761 PMC9846462

[B37] Moretti J. Terstege D. J. Poh E. Z. Epp J. R. Rodger J. (2022). Low intensity repetitive transcranial magnetic stimulation modulates brain-wide functional connectivity to promote anti-correlated c-Fos expression. *Sci. Rep.* 12:20571. 10.1038/s41598-022-24934-8 36446821 PMC9708643

[B38] Ortiz-Prado E. Reascos M. S. Núñez D. F. Pazmiño J. C. Gallardo M. Salazar N. et al. (2025). Detrimental effects of hypoxia on cognitive function: adaptations, challenges, and resilience in andean populations. *J. Racial Ethn. Health Disparities* 12 3569–3575. 10.1007/s40615-025-02609-0 40864381

[B39] Reuter-Lorenz P. A. Cappell K. A. (2008). Neurocognitive aging and the compensation hypothesis. *Curr. Direct. Psychol. Sci.* 17 177–182. 10.1111/j.1467-8721.2008.00570.x

[B40] Rossi S. Hallett M. Rossini P. M. Pascual-Leone A. Safety of Tms Consensus Group. (2009). Safety, ethical considerations, and application guidelines for the use of transcranial magnetic stimulation in clinical practice and research. *Clin. Neurophysiol.* 120 2008–2039. 10.1016/j.clinph.2009.08.016 19833552 PMC3260536

[B41] Sasaki N. Mizutani S. Kakuda W. Abo M. (2013). Comparison of the effects of high- and low-frequency repetitive transcranial magnetic stimulation on upper limb hemiparesis in the early phase of stroke. *J. Stroke Cerebrovasc. Dis*. 22 413–418. 10.1016/j.jstrokecerebrovasdis.2011.10.004 22177936

[B42] Su R. Jia S. Zhang N. Wang Y. Li H. Zhang D. et al. (2024). The effects of long-term high-altitude exposure on cognition: a meta-analysis. *Neurosci. Biobehav. Rev.* 161:105682. 10.1016/j.neubiorev.2024.105682 38642865

[B43] Tang A. D. Bennett W. Bindoff A. D. Bolland S. Collins J. Langley R. C. et al. (2021). Subthreshold repetitive transcranial magnetic stimulation drives structural synaptic plasticity in the young and aged motor cortex. *Brain Stimul*. 14 1498–1507. 10.1016/j.brs.2021.10.001 34653682

[B44] Taylor J. L. Bhatt P. Hernandez B. Iv M. Adamson M. M. Heath A. et al. (2025). Network-targeted transcranial magnetic stimulation (TMS) for mild cognitive impairment (MCI). *Neuroimage Clin*. 47:103819. 10.1016/j.nicl.2025.103819 40513355 PMC12192757

[B45] Volpe U. Mucci A. Bucci P. Merlotti E. Galderisi S. Maj M. (2007). The cortical generators of P3a and P3b: a LORETA study. *Brain Res. Bull.* 73 220–230. 10.1016/j.brainresbull.2007.03.003 17562387

[B46] Walla P. (2025). The power of time: editorial on the advantages of electroencephalography (EEG) and event-related potentials (ERPs) in affective and cognitive neuroscience. *Brain Sci*. 15:1054. 10.3390/brainsci15101054 41154150 PMC12562382

[B47] Wang K. Li Q. Zheng Y. Wang H. Liu X. (2014). Temporal and spectral profiles of stimulus-stimulus and stimulus-response conflict processing. *Neuroimage* 89 280–288. 10.1016/j.neuroimage.2013.11.045 24315839

[B48] Wang L. Sang L. Cui Y. Li P. Qiao L. Wang Q. et al. (2022). Effects of acute high-altitude exposure on working memory: a functional near-infrared spectroscopy study. *Brain Behav*. 12:e2776. 10.1002/brb3.2776 36321845 PMC9759148

[B49] Wang Y. Wang L. Ni X. Jiang M. Zhao L. (2024). Efficacy of repetitive transcranial magnetic stimulation with different application parameters for post-stroke cognitive impairment: a systematic review. *Front. Neurosci*. 18:1309736. 10.3389/fnins.2024.1309736 38567284 PMC10985147

[B50] Yüce A. E. Yaman I. Gündoğdu S. (2026). EEG/MEG-based biomarkers of cognitive training effects in schizophrenia: a systematic review. *Psychiatry Res. Neuroimaging* 357:112112. 10.1016/j.pscychresns.2025.112112 41558276

[B51] Zhong Z. Zhou S. Xiang B. Wu Y. Xie J. Li P. (2021). Association of peripheral plasma neurotransmitters with cognitive performance in chronic high-altitude exposure. *Neuroscience* 463 97–107. 10.1016/j.neuroscience.2021.01.031 33540052

