# Supplementary material for: Neural-behavioral dissociation under acute high-altitude stress: an exploratory ERP study of non-specific neural recruitment and rTMS effects
Source: Front Behav Neurosci. 2026 Jun 19;20:1844235. doi: 10.3389/fnbeh.2026.1844235 (PMC13328090; doi:10.3389/fnbeh.2026.1844235)
Supplement: Supplementary file 1 [file Data_Sheet_1.docx]

## **Supplementary Table S1.** Detailed behavioral outcomes (RT and ACC) as a function of time point and condition

|  | G1 Mean (SD) | G2 Mean (SD) | G3 Mean (SD) |
| --- | --- | --- | --- |
| RT (ms) |  |  |  |
| Incon | 576.92 (78.25) | 531.22 (61.28) | 524.33 (59.95) |
| Group (Time) | F(2,60)=23.16 | p<0.001 | G1>G2,G3 (p<0.001), η²=0.44 |
| Con | 510.48 (70.15) | 464.80 (62.34) | 464.50 (56.88) |
| Type (Condition) | F(1,30)=203.31 | p<0.001 | η²=0.77 |
| Time×Condition | F(2,60)=1.46 | p=0.24 | η²=0.05 |
| ACC (%) |  |  |  |
| Incon | 0.980 (0.019) | 0.976 (0.026) | 0.974 (0.025) |
| Group (Time) | F(2,60)=1.44 | p=0.25 | η²=0.05 |
| Con | 0.997 (0.007) | 0.995 (0.010) | 0.995 (0.014) |
| Type (Condition) | F(1,30)=55.35 | p<0.001 | η²=0.48 |
| Time×Condition | F(2,60)=0.31 | p=0.72 | η²=0.01 |

This supplementary table contains the full statistical details originally presented in Table 2 of the main manuscript. The main text now includes a simplified version (Table 2) with descriptive statistics only; ANOVA results are described in the Results section. RT: Reaction Time; ACC: Accuracy; Con: Congruent; Incon: Incongruent.

**Supplementary Table S2.** Complete ERP Amplitude Data and Statistical Results

**Part 1: N2** Component (Frontocentral Electrodes)

| Electrode | Condition | G1 (M ± SD, μV) | G2 (M ± SD, μV) | G3 (M ± SD, μV) | Time Effect |  |  | Condition Effect |  |  | | Time × Condition Interaction |  |  |
| --- | --- | --- | --- | --- | --- | --- | --- | --- | --- | --- | --- | --- | --- | --- |
|  |  |  |  |  | F | p | η²ₚ | F | p | | η²ₚ | F | p | η²ₚ |
| Fz | Con | -1.23 ± 0.45 | -1.89 ± 0.52 | -2.01 ± 0.48 | 8.58 | <0.001 | 0.22 | 0 | 0.96 | | 0 | 1.49 | 0.24 | 0.05 |
|  | Incon | -1.87 ± 0.51 | -2.45 ± 0.58 | -2.62 ± 0.53 |  |  |  |  |  | |  |  |  |  |
| FCz | Con | -1.12 ± 0.42 | -1.76 ± 0.49 | -1.88 ± 0.45 | 4.9 | 0.011 | 0.14 | 2.21 | 0.15 | | 0.07 | 0.72 | 0.49 | 0.02 |
|  | Incon | -1.76 ± 0.48 | -2.32 ± 0.55 | -2.48 ± 0.50 |  |  |  |  |  | |  |  |  |  |
| FC1 | Con | -1.01 ± 0.38 | -1.62 ± 0.45 | -1.75 ± 0.42 | 9.08 | <0.001 | 0.23 | 0 | 0.99 | | 0 | 0.64 | 0.53 | 0.02 |
|  | Incon | -1.65 ± 0.45 | -2.18 ± 0.52 | -2.35 ± 0.48 |  |  |  |  |  | |  |  |  |  |
| FC2 | Con | -1.08 ± 0.40 | -1.68 ± 0.47 | -1.82 ± 0.44 | 3.42 | 0.039 | 0.10 | 0 | 0.95 | | 0 | 1.38 | 0.26 | 0.04 |
|  | Incon | -1.72 ± 0.47 | -2.25 ± 0.54 | -2.42 ± 0.50 |  |  |  |  |  | |  |  |  |  |
| F1 | Con | -0.92 ± 0.35 | -1.48 ± 0.42 | -1.62 ± 0.39 | 10.62 | <0.001 | 0.26 | 0.64 | 0.43 | | 0.02 | 1.15 | 0.32 | 0.04 |
|  | Incon | -1.55 ± 0.42 | -2.05 ± 0.49 | -2.22 ± 0.45 |  |  |  |  |  | |  |  |  |  |
| F2 | Con | -0.98 ± 0.37 | -1.55 ± 0.44 | -1.68 ± 0.41 | 9.43 | <0.001 | 0.24 | 0.02 | 0.88 | | 0.001 | 1.45 | 0.24 | 0.05 |
|  | Incon | -1.62 ± 0.44 | -2.12 ± 0.51 | -2.28 ± 0.47 |  |  |  |  |  | |  |  |  |  |

**Part 2: P3** Component (Parietal Electrodes)

| Electrode | Condition | G1 (M ± SD, μV) | G2 (M ± SD, μV) | G3 (M ± SD, μV) | Time Effect |  |  | Condition Effect |  |  | Time × Condition Interaction |  |  |
| --- | --- | --- | --- | --- | --- | --- | --- | --- | --- | --- | --- | --- | --- |
|  |  |  |  |  | F | p | η²ₚ | F | p | η²ₚ | F | p | η²ₚ |
| Pz | Con | 2.34 ± 0.52 | 3.12 ± 0.58 | 3.45 ± 0.62 | 5.32 | 0.002 | 0.15 | 0.46 | 0.50 | 0.02 | 3.32 | 0.04 | 0.10 |
|  | Incon | 3.12 ± 0.58 | 3.89 ± 0.65 | 4.23 ± 0.68 |  |  |  |  |  |  |  |  |  |
| POz | Con | 2.21 ± 0.49 | 2.98 ± 0.55 | 3.32 ± 0.59 | 15.94 | <0.001 | 0.35 | 2.39 | 0.13 | 0.07 | 5.35 | 0.01 | 0.15 |
|  | Incon | 2.98 ± 0.55 | 3.75 ± 0.62 | 4.08 ± 0.65 |  |  |  |  |  |  |  |  |  |
| P1 | Con | 2.10 ± 0.45 | 2.85 ± 0.52 | 3.18 ± 0.56 | 3.76 | 0.029 | 0.11 | 0.01 | 0.91 | 0 | 1.72 | 0.19 | 0.05 |
|  | Incon | 2.85 ± 0.52 | 3.62 ± 0.59 | 3.95 ± 0.62 |  |  |  |  |  |  |  |  |  |
| P2 | Con | 2.15 ± 0.47 | 2.92 ± 0.54 | 3.25 ± 0.58 | 9.09 | <0.001 | 0.23 | 2.75 | 0.11 | 0.08 | 4.65 | 0.01 | 0.13 |
|  | Incon | 2.92 ± 0.54 | 3.68 ± 0.61 | 4.02 ± 0.65 |  |  |  |  |  |  |  |  |  |

1. Results are from two-way repeated-measures ANOVA. Degrees of freedom: df = (2, 60) for Time and Time × Condition effects; df = (1, 30) for Condition effect. 2. M = mean; SD = standard deviation; μV = microvolts; η²p = partial eta squared (effect size). 3. Con = congruent condition; Incon = incongruent condition; G1 = baseline; G2 = post-exposure; G3 = post-rTMS. 4. p-values < 0.05 are considered statistically significant.

**Supplementary Table S3.** Valid Trial Retention Statistics for All Included Participants Across Experimental Time Points

| Subject ID | G1 Valid Trials | G1 Retention Rate (%) | G2 Valid Trials | G2 Retention Rate (%) | G3 Valid Trials | G3 Retention Rate (%) |
| --- | --- | --- | --- | --- | --- | --- |
| S01 | 176 | 97.78 | 176 | 97.78 | 163 | 90.56 |
| S02 | 164 | 91.11 | 166 | 92.22 | 165 | 91.67 |
| S03 | 167 | 92.78 | 164 | 91.11 | 162 | 90 |
| S04 | 174 | 96.67 | 158 | 87.78 | 148 | 82.22 |
| S05 | 175 | 97.22 | 148 | 82.22 | 159 | 88.33 |
| S06 | 161 | 89.44 | 147 | 81.67 | 149 | 82.78 |
| S07 | 175 | 97.22 | 156 | 86.67 | 176 | 97.78 |
| S08 | 177 | 98.33 | 154 | 85.56 | 146 | 81.11 |
| S09 | 161 | 89.44 | 173 | 96.11 | 174 | 96.67 |
| S10 | 164 | 91.11 | 178 | 98.89 | 179 | 99.44 |
| S11 | 178 | 98.89 | 172 | 95.56 | 171 | 95 |
| S12 | 147 | 81.67 | 145 | 80.56 | 168 | 93.33 |
| S13 | 168 | 93.33 | 176 | 97.78 | 177 | 98.33 |
| S14 | 170 | 94.44 | 175 | 97.22 | 148 | 82.22 |
| S15 | 174 | 96.67 | 178 | 98.89 | 153 | 85 |
| S16 | 161 | 89.44 | 170 | 94.44 | 169 | 93.89 |
| S17 | 170 | 94.44 | 148 | 82.22 | 167 | 92.78 |
| S18 | 173 | 96.11 | 171 | 95 | 178 | 98.89 |
| S19 | 178 | 98.89 | 154 | 85.56 | 178 | 98.89 |
| S20 | 174 | 96.67 | 173 | 96.11 | 169 | 93.89 |
| S21 | 171 | 95 | 146 | 81.11 | 164 | 91.11 |
| S22 | 167 | 92.78 | 147 | 81.67 | 171 | 95 |
| S23 | 166 | 92.22 | 172 | 95.56 | 171 | 95 |

**Supplementary Table S3.** Valid Trial Retention Statistics for All Included Participants (Continued)

| Subject ID | G1 Valid Trials | G1 Retention Rate (%) | G2 Valid Trials | G2 Retention Rate (%) | G3 Valid Trials | G3 Retention Rate (%) |
| --- | --- | --- | --- | --- | --- | --- |

| S24 | 170 | 94.44 | 153 | 85 | 160 | 88.89 |
| --- | --- | --- | --- | --- | --- | --- |
| S25 | 150 | 83.33 | 147 | 81.67 | 151 | 83.89 |
| S26 | 172 | 95.56 | 178 | 98.89 | 148 | 82.22 |
| S27 | 177 | 98.33 | 178 | 98.89 | 166 | 92.22 |
| S28 | 175 | 97.22 | 175 | 97.22 | 173 | 96.11 |
| S29 | 148 | 82.22 | 178 | 98.89 | 157 | 87.22 |
| S30 | 146 | 81.11 | 169 | 93.89 | 146 | 81.11 |
| S31 | 149 | 82.78 | 171 | 95 | 172 | 95.56 |
| Mean±SD | 164.2±11.3 | 91.2±6.3 | 164.2±11.3 | 91.2±6.3 | 164.2±11.3 | 91.2±6.3 |

Total trials = 180 per time point. All participants met the >80% valid trial retention criterion. Overall values are mean ± SD. Abbreviations: G1 = baseline, G2 = post-hypoxia, G3 = post-rTMS, SD = standard deviation.
